# Risk factors for serious outcomes associated with influenza illness in high‐ versus low‐ and middle‐income countries: Systematic literature review and meta‐analysis

**DOI:** 10.1111/irv.12504

**Published:** 2017-12-02

**Authors:** Brenda L. Coleman, Shaza A. Fadel, Tiffany Fitzpatrick, Sera‐Melisa Thomas

**Affiliations:** ^1^ Sinai Health System, Infectious Disease Epidemiology Research Unit University of Toronto Toronto ON Canada

**Keywords:** hospitalization, income, influenza, mortality, risk factors

## Abstract

**Aim:**

To determine factors associated with a serious outcome (hospital admission or severe outcome: critical care or death) and associated with illness caused by laboratory‐confirmed influenza, with a specific interest in low‐ and middle‐income countries (LMIC).

**Method:**

Databases were searched on 11 March 2016 for reports of influenza and factors associated with mortality or morbidity in humans, with no language restrictions. Pooled risks were estimated using random‐effects models.

**Results:**

Despite the heterogeneity of results across studies, known risk factors for serious disease were associated with both hospital admission and severe outcomes (critical care and/or death). In LMIC, but not in high income countries (HIC), pregnant women, people with HIV/AIDS and children < 5 years old (compared with older children) were at increased risk of a severe outcome. Also, although all patients with neurological conditions were at higher risk of severe outcomes than those without, children were at higher risk than adults and children who lived in a LMIC were at significantly higher risk than those living in HIC. Adults were more likely than children to suffer a severe outcome if they had diabetes or a hematologic condition, were obese or had liver disease. Asthma is a risk factor for hospital admission but not for severe outcomes.

**Conclusion:**

Known risk factors for serious disease remain important predictors of hospital admission and severe outcomes with few differences between HIC and LMIC countries. These differences likely reflect differences in health‐seeking behaviours and health services, but high heterogeneity between studies limits conclusions about the effect size.

## 1 INTRODUCTION

Influenza viruses cause 250 000 to 500 000 deaths annually worldwide.^1^ Defining people who are at higher risk of serious outcomes when ill with influenza will assist public health officials and clinicians to prioritize vaccination, treatment and hospital admission policy and practice.

Recommendations for vaccination vary by country but are based on previously identified risk factors for serious outcomes in people ill with influenza. The majority of low‐ and middle‐income countries (LMIC) lack the capacity for extensive influenza surveillance and some do not have national policies for seasonal vaccination.^2^ In high‐income countries (HIC), it is generally recommended that vaccination against influenza occurs for children aged 6‐59 months; older adults; people with chronic pulmonary, cardiovascular, renal, hepatic, neurologic, hematologic or metabolic disorders; persons who are immunosuppressed; women who are, or will be, pregnant during the influenza season; children who are at increased risk of Reye's syndrome; residents of nursing homes and other long‐term care facilities; and persons who are morbidly obese (body mass index [BMI] of ≥40).^3^, ^4^, ^5^, ^6^, ^7^ These terms provided the starting point for the factors investigated in this review; others were included if they were identified by several different researchers as being related to serious outcomes in people with influenza illness.

The World Health Organization (WHO) commissioned this systematic review of the literature to determine the factors associated with serious outcomes following an illness caused by seasonal influenza, with a focus on LMIC. We conducted a meta‐analysis to compare and contrast outcomes by risk factor for HIC and LMIC for laboratory‐confirmed influenza.

## 2 METHODS

The published literature was searched from the database's inception to 11 March 2016 using EMBASE, Cochrane Central Register of Controlled Trials, MEDLINE, Web of Science and Global Health‐Public Health EBSCO without language exclusion. The search was limited to humans and included terms covering influenza or severe acute respiratory infection and outcomes of mortality, hospitalization, pneumonia, morbidity, critical illness or respiratory support with information on risk factors (see supplementary material). Opinion and editorial papers, case reports and book chapters were excluded. The reference lists of relevant studies and grey literature sources were examined to 21 April 2016. Corresponding authors were contacted for data needed for the meta‐analyses. The review was registered with PROSPERO (#42016040014).^8^

Two independent reviewers screened publications (title, abstract and text) to include only human influenza and those with risk factors associated with, and including, serious outcomes. The 2015 World Bank country classifications^9^ were used to categorize data by income groups (ie, LMIC and HIC). Two reviewers (SAF, SMT, TF or BLC) independently assessed each full‐text version of English language and translated articles. Articles written in Chinese, Korean and Japanese were assessed and data were abstracted by individuals with the same mother tongue, while other non‐English articles were translated using translation software.^10^ An assessment of risk of bias was completed, at the study level, by each reviewer using the Newcastle‐Ottawa Scale for cohort, case‐control or cross‐sectional studies.^11^, ^12^ Studies that scored two points or less on any of the risk of bias scales were excluded. Publications restricted to the description of age and/or sex only and those without comparison groups were not included. Studies limited to a comparison of high‐risk populations to one another (ie, ICU patients or patients with cancer, diabetes or pneumonia) and health care‐associated cases were excluded as they do not represent the general population. If more than one publication was identified as using the same patients, only the more inclusive (eg, additional seasons) or more relevant (ie, for this review) was included. All discrepancies were settled through discussion and re‐review of the study. The flow diagram follows PRISMA guidelines.^13^

Outcomes include the following: (i) all‐cause mortality; (ii) admission to an intensive care unit (ICU) with/without mechanical ventilation; (iii) a “critical” outcome (ie, ICU admission and/or death); and (iv) hospital admission for people with laboratory‐confirmed influenza (polymerase chain reaction (PCR), culture, direct fluorescent antibody (DFA), enzyme immunoassay (EIA) or rapid antigen (RAT) testing). To synthesize data, a derived variable, “severe” outcome, includes data for one of an author‐defined critical outcome (ICU and/or death), ICU admission or death. If a study provided data on more than one of these outcomes, this derived variable includes only one, with priority as listed above.

To help reduce heterogeneity of estimates, we restricted the outcomes to those for people ill with laboratory‐confirmed influenza and hospitalized cases were used as comparators for ICU admissions and deaths, while influenza‐positive non‐hospitalized cases were used as the comparison for hospital admissions. Risk factors were categorized as defined by the original authors. When authors disaggregated reported outcomes within chosen categories (eg, neurological and neuromuscular conditions), the more inclusive of the subcategories was used in the meta‐analysis.

Pooled relative risks were estimated using random‐effects models to account for various study designs, testing methods and populations. The DerSimonian and Laird method of statistical estimation of effect size and the Mantel‐Haenszel estimate of heterogeneity were used to account for low event rates and/or study sizes using Stata v14.2 with zero cells in pooled relative risk (pRR) estimates only (not odds ratios) being given a value of 0.1.^14^ The degree of statistical heterogeneity was assessed using the I^2^ index. Readers are advised that meta‐analytic estimates produced when the number of studies is small should be interpreted with caution.

## 3 RESULTS

**Figure 1:**
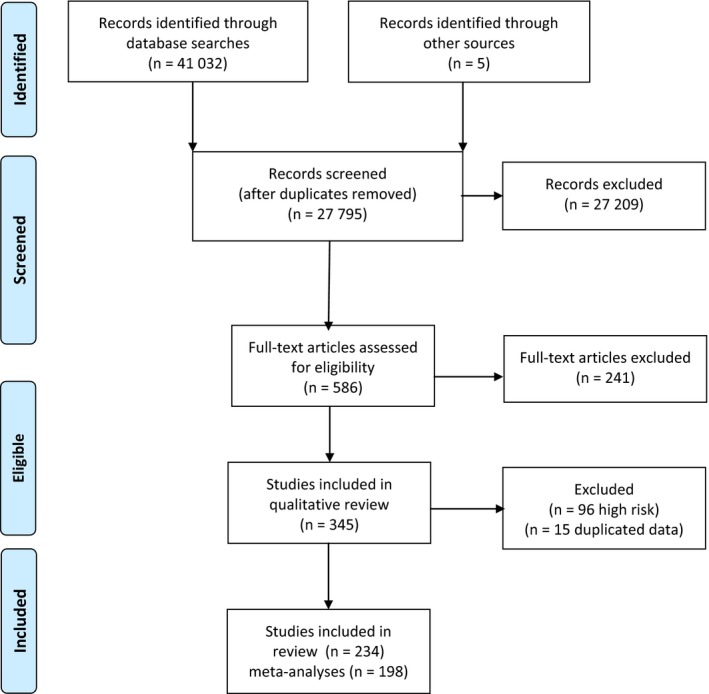
Flow diagram for article screening and selection

Of the 27 795 articles identified, 345 were eligible for review. Ninety‐six were excluded because they included only high‐risk patients and 15 articles were excluded due to data replicated in other publications leaving 234 for the full review. The 198 studies presented in the meta‐analyses included 72 restricted to adults, 37 to children (generally <20 years old but as defined by the authors) and 103 for all ages. Studies were mainly conducted during the 2009 pandemic (N = 158), with 35 from non‐pandemic, and 12 from both pandemic and non‐pandemic seasons. Most (N = 141) studies occurred in HIC representing 36 different countries, while 57 studies from LMIC countries (37 upper middle, 19 lower middle, 1 low income) represented 17 different countries. Although only 2 studies from LMIC were for pre‐pandemic seasons, there was no statistical difference by country income level and year of data collection (*P* = .79), with 9.1% of LMIC and 10.7% of HIC data collected post‐pandemic. No study was excluded due to high risk of bias. Thirty‐six other studies were used only for the review as they did not provide individual‐level data for the meta‐analysis (see Figure 1).

### 3.1 Hospital admission

In HIC, higher rates of hospital admission were reported for children <5 years old compared with older children (5‐19 years old), young adults (20‐64 years of age) or older adults (65 years and older) with pooled relative risks (pRR) of 2.36, 4.54 and 2.53, respectively (Table S1a‐c). In comparison, the one study from a LMIC, conducted in India, reported a lower rate of hospital admission of children <5 years of age compared with older children, young and older adults (pRR 0.84, 0.55 and 0.36, respectively).^15^ Young adults were significantly less likely to be admitted to hospital than older adults in both HIC and LMIC countries and in both pandemic and non‐pandemic seasons. There were no differences in the risk of hospital admission by sex, with a pRR for males vs females of 1.07 (95% confidence interval 0.98‐1.16; I^2^ = 74.0%; n = 26).

People with one or more underlying conditions (as defined by the study authors) were at more than twice the risk of hospital admission as people without a comorbidity (pRR 2.25; 1.81‐2.80; I^2^ = 94.7%; N = 21). This estimate was similar for both HIC and LMIC and for pandemic and seasonal estimates; however, the association was lower for children (pRR 1.65; 1.30‐2.09; I^2^ = 81.3%; n = 7) than for adults (pRR 3.00; 2.06‐4.35; I^2^ = 69.5%; n = 5). This was due, in part, to the increased risk of hospital admission for adults with diabetes mellitus compared to those without (pRR 2.37; 1.98‐2.84; I^2^ = 86.3%; n = 17), while there was no difference for children with and without diabetes (1.08; 0.39‐3.03; I^2^ = 85.7%; n = 3). The risk of hospital admission for diabetes was similar for HIC and LMIC and for pandemic and non‐pandemic seasons. On the other hand, the estimates for the risk of hospital admission were similar for adults and children, for HIC and LMIC, and during pandemic and non‐pandemic seasons for people with versus those without: neurological and neuromuscular conditions (pRR 2.09; 1.73‐2.54; I^2^ = 86.6%; n = 16), malignancies (pRR 2.05; 1.66‐2.53; I^2^ = 55.6%; n = 10), immune‐suppressing conditions (pRR 2.03; 1.47‐2.80; I^2^ = 91.5%; n = 15), renal disease (pRR 2.03; 1.48‐2.77; I^2^ = 93.3%; n = 18), cardiac conditions (pRR 1.98; 1.64‐2.38; I^2^ = 89.0%; n = 21), chronic lung disease (pRR 1.96; 1.73‐2.23; I^2^ = 80.3%; n = 21), hematologic conditions (pRR 1.85; 1.16‐2.94; I^2^ = 94.3%; n = 5), obesity (pRR 1.82 (1.48‐2.24; I^2^ = 84.9%; n = 15), metabolic conditions (1.78; 1.14‐2.77; I^2^ = 0; n = 2), liver disease (pRR 1.67; 1.21‐2.31; I^2^ = 88.2%; n = 9), pregnancy (pRR 1.56; 1.16‐2.09; I^2^ = 95.5%; n = 10), asthma (pRR 1.56; 1.02‐2.40; I^2^ = 96.2%; n = 11) and tobacco smoking (pRR 1.24; 1.07‐1.43; I^2^ = 61.5%; n = 10). See also Table S2a‐j.

People with HIV/AIDS were often categorized together with people with other immunosuppressive conditions. For those studies from HIC that separated them, the pRR of hospital admission of people ill with influenza and with HIV/AIDS was no higher than for people without HIV/AIDS (pRR 0.94; 0.37‐2.10; I^2^ = 87.4%; n = 3). This is consistent with the adjusted OR of 1.31 (0.39‐4.37) reported by one (included) study, a matched case‐control study conducted in Spain during the 2009 H1N1 pandemic.^16^ In contrast, one study conducted in a LMIC reported significantly higher rates of hospital admission for HIV‐infected people, with the relative risk ranging from 4.2 to 7.5 by season (2009‐2011) and also varying by age group, with the highest risk among 25‐ to 44‐year‐old adults.^17^ There were also few studies that separated people with tuberculosis (TB) from other diseases; among those that did, data were not provided for latent infection versus active TB disease and, in fact, no studies provided data specific to the risk of hospital admission for people with TB.

In the review of studies that provided estimates adjusted for the presence of other factors known to impact the risk of hospital admission for people ill with influenza, we found similarly heterogeneous estimates. There were no adjusted estimates that were significantly and consistently (across studies) outside the confidence bounds of the estimated pRR from this meta‐analysis.^18^, ^19^, ^20^, ^21^, ^22^, ^23^, ^24^, ^25^, ^26^

### 3.2 Severe outcome (intensive care and/or death)

The risk of having a severe outcome (ICU admission and/or death) was lower for young children (<5 years) than for older children (5‐19 years of age) in HIC (pRR 0.74; 0.59‐0.92; I^2^ = 65.7%; n = 15). However, young children in LMIC were more likely than older children to have a severe outcome (pRR 1.52; 1.09‐2.13; I^2^ = 13.0%; n = 8), with no difference between pandemic and non‐pandemic seasons. Young children in both HIC and LMIC were significantly less likely to suffer a severe outcome than young adults (pRR 0.37; 0.25‐0.55; I^2^ = 93.4%; n = 19) or older adults (pRR 0.25; 0.11‐0.36; I^2^ = 79.0%; n = 17). Similarly, older children were less likely to have severe outcomes when ill with influenza than young adults (pRR 0.40; 0.25‐0.64; I^2^ = 95.0%; n = 18) or older adults (pRR 0.40; 0.30‐0.54; I^2^ = 65.4%; n = 15). Similarly, young adults were less likely than older adults (pRR 0.69; 0.57‐0.84; I^2^ = 84.5%; n = 38) to have suffered severe outcomes, with no differences in estimates for HIC and LMIC or during pandemic and non‐pandemic seasons. There was a small increased risk of a severe outcome for male compared with female children (pRR 1.20; 1.02‐1.40; I^2^ = 0; n = 12), which was not observed for adult males compared with adult females (pRR 1.04; 0.97‐1.11; I^2^ = 36.5%; n = 32).

Patients with one or more underlying conditions were twice as likely to suffer severe outcomes when ill with influenza as patients without (pRR 1.94; 1.64‐2.29; I^2^ = 93.4%; n = 87), with no difference by country income, between adults and children, or for pandemic and non‐pandemic influenza seasons (Figures S1‐3). Patients more likely to suffer severe outcomes included those with malignancies (1.81; 1.35‐2.23; I^2^ = 83.4%; n = 33), immune‐suppressing conditions (pRR 1.51; 1.23‐1.85; I^2^ = 89.3%; n = 63), renal conditions (pRR 1.74; 1.45‐2.09; I^2^ = 80.6%; n = 48), cardiac conditions (pRR 1.91; 1.64‐2.24; I^2^ = 90.9%; n = 77), lung conditions (1.53; 1.33‐1.76; I^2^ = 89.0%; n = 84), tobacco smoking (pRR 1.46; 1.25‐1.69; I^2^ = 62.4%; n = 30) or TB disease (pRR 3.22 (1.98, 5.26; I^2^ = 16.4%; n = 5). Of note, Cohen et al^27^ reported that the unadjusted odds of mortality for South African patients with a TB diagnosis of 2.7 (0.6‐13.4) were lower than for those being treated for TB disease (OR 3.9; 1.9‐7.0). Conversely, there was no increased risk of severe outcomes for patients with asthma (pRR 0.86; 0.72‐1.04; I^2^ = 81.4%; n = 58). All of the above estimates were similar for HIC and LMIC as well as for pandemic/non‐pandemic seasons and patient's age (adult/child).

Pregnant women living in LMIC were at 66% increased risk of severe outcomes compared with other patients ill with influenza (pRR 1.66; 1.20‐2.31; I^2^ = 91.3%; n = 31); however, there was no increased risk for pregnant women living in HIC (pRR 0.80; 0.60‐1.05; I^2^ = 73.1%; n = 42). No differences were observed for the risk of severe outcomes in pregnant women when comparing studies from pandemic and non‐pandemic seasons. Similarly, the pRR of a severe outcome for patients infected with HIV was not significantly higher for people without HIV, for those living in HIC (1.18; 0.84‐1.67; I^2^ = 23.7%; n = 6) but was higher for people living in LMIC (2.17; 1.29‐1.91; I^2^ = 0; n = 3). In support of this finding for LMIC, the adjusted odds of death were higher for HIV‐positive patients than uninfected patients living in South Africa (2.9; 1.1, 7.8).^27^

The risk of a severe outcome for patients with influenza and pre‐existing neurological conditions was higher among children (pRR 3.08; 2.47‐3.85; I^2^ = 69.4%; n = 22) than adults (1.67; 1.09‐2.58; I^2^ = 82.2%; n = 13). Although the number of studies in LMIC was low, it appears that children with neurological conditions living in LMIC (pRR 5.07; 3.59‐7.16; I^2^ = 0; n = 2) had higher risk ratio than children from HIC (pRR 2.87; 2.31‐3.58; I^2^ = 64.5%; n = 20) (Figure S4); no difference was detected for adults by country income level. Children with metabolic conditions were also at higher risk of severe outcomes (pRR 2.03; 1.41‐2.92; I^2^ = 0; n = 5) than adults (pRR 1.17; 1.02‐1.34; I^2^ = 0; n = 3), with no difference in estimates for LMIC and HIC.

Conversely, adults with diabetes mellitus were at higher risk of a severe outcome than adults without (pRR 1.23; 1.05‐1.43; I^2^ = 34.8%; n = 24), while there was no difference among children (pRR 1.28; 0.44‐3.74; I^2^ = 0; n = 2). Obese adults were also at higher risk of severe outcomes than healthy weight adults (pRR 1.40; 1.01‐1.95; I^2^ = 89.3%; n = 24); there was no increased risk for obese children (pRR 0.91; 0.47‐1.74; I^2^ = 53.2%; n = 8). Similarly, adults with hematologic conditions were at higher risk of severe outcomes than adults without (pRR 2.15; 1.62‐2.85; I^2^ = 0; n = 3), while the risk was not higher for children (pRR 0.60; 0.17‐2.13; I^2^ = 88.3%; n = 9). And, although adults with liver disease were at higher risk of severe outcomes than those without (2.01; 1.45‐2.79; I^2^ = 52.1%; n = 11), there was no difference in children (pRR 0.47; 0.13‐1.71; I^2^ = 0; n = 2). No differences were detected by country income level or during pandemic/non‐pandemic influenza seasons.

In the review of studies that provided estimates adjusted for the presence of other factors known to impact the risk of severe outcomes for people ill with influenza, we found that the risk estimate was heterogeneous and not consistently greater or less than the pRR estimated with this meta‐analysis.^21^, ^27^, ^28^, ^29^, ^30^, ^31^, ^32^, ^33^, ^34^, ^35^, ^36^, ^37^, ^38^, ^39^, ^40^, ^41^, ^42^, ^43^, ^44^, ^45^, ^46^, ^47^, ^48^, ^49^, ^50^, ^51^, ^52^, ^53^, ^54^, ^55^

## 4 DISCUSSION

This systematic literature review and meta‐analysis found few differences between HIC and LMIC for the risk of hospital admission or severe outcomes (ICU and/or death) for people ill with laboratory‐confirmed influenza A(H1N1), A(H3N2) or B. There were, however, four differences worthy of note. First, young children (<5 years old) living in HIC had a similar risk of death if hospitalized with influenza as older children (5‐19 years), while in LMIC, the risk of severe outcomes was 50% higher for the younger age group. Note, however, that children of any age were at significantly lower risk of severe outcomes than younger or older adults in both LMIC and HIC. Second, pregnant women in HIC had a similar risk of severe outcomes as non‐pregnant women, while those living in LMIC were at 66% higher risk of a severe outcome than their non‐pregnant peers. Also, in the few studies that separated patients with HIV/AIDS from others with immune‐suppressing conditions, those living in HIC had a similar risk of a severe outcome as people without the disease, while patients in LMIC had more than twice the risk of needing intensive care and/or dying. Finally, children in LMIC with neurological conditions had 5 times the risk of a severe outcome, while those living in a HIC had 3 times the risk compared to children without neurological conditions. These findings likely reflect differences in health‐seeking behaviour and access to health care.^56^, ^57^

This meta‐analysis confirms the association between influenza illness and increased risk of both hospital admission and severe outcomes for known influenza risk factors. Although there were few differences in the risk of hospital admission by patient age, the risk of a severe outcome was higher for adults than children with diabetes mellitus, obesity (BMI ≥30 kg/m^2^) or liver conditions when compared to those without. This is not unexpected given that the mortality rates associated with diabetes and liver disease as well as with conditions linked to obesity, such as heart disease, increase significantly by age.^58^, ^59^ Conversely, children with neurological or metabolic conditions had a higher risk of severe outcome than adults with these conditions, when compared to children or adults, respectively, without them. The broad, and generally unreported, range of neurological and metabolic conditions included in each publication's definition of the factor makes interpretation of these findings fraught. In this review, we did not include diabetes within the metabolic conditions category unless the author did so, which was infrequent. As many non‐diabetes–related metabolic conditions cause significant morbidity and mortality during childhood, it may increase the risk of severe outcomes for children versus adults. It is possible that the same issue is occurring with neurological conditions, another broad category. Finally, although people ill with influenza and the underlying condition of asthma were more likely to be hospitalized than people without asthma, they were not at increased risk of a severe outcome. This was not found for other chronic respiratory conditions, which included chronic obstructive lung disease, for which there was a higher risk of hospital admission and a higher risk of severe outcomes. This is explained by the fact that although asthma is a common underlying condition that is exacerbated by respiratory illnesses, it is not a common cause of death. In the USA, for example, the mortality rate for chronic respiratory diseases was 47.2 per 100 000 in 2013, of which asthma contributed just over 2% of cases (1.1 per 100 000).

### 4.1 Limitations

The primary limitation of this review is the high heterogeneity scores for results of many of these estimates. Although the heterogeneity is not unexpected given the observational nature of the studies, it does limit the ability to accurately estimate the size of the effects. Several factors impact the heterogeneity including myriad definitions employed for each risk factor, the comparison groups used, different influenza type/subtypes and the age of people included in each study. Heterogeneity may also have been introduced through social and cultural factors, particularly those affecting health care‐seeking behaviours or the hospital‐to‐hospital and country‐to‐country variability in criteria for admission to hospital or intensive care and influenza testing methods, protocols and practices. Given the high I^2^ values on many estimates, readers are encouraged to use caution if trying to interpret the size of the effects. A second limitation is the lack of data for many of the risk factors. Estimates produced from a small number of studies must be interpreted with caution. This limitation is especially apparent when estimating effects in LMIC.

Although most studies compared the ages of people hospitalized or suffering severe outcomes when ill with influenza, the myriad groupings significantly impeded the direct comparison of results by age group. We also chose not to abstract data from studies with age or sex as the only reported risk factor. Another limitation was the inability to separate the risk for adults and children in many studies as the authors did not stratify their analyses by age. Similarly, although some studies have noted differences in severe outcomes by influenza type and/or subtype,^60^ we were unable to assess the impact of such differences. Finally, the range of definitions for “severe” outcomes was also a limiting factor. For the meta‐analysis, we chose to include only admission to ICU and/or death. Many authors included pneumonia in their definition of a severe outcome but, due to the broad range of severity of illness and range of criteria used to diagnose pneumonia, we chose not to include it.

## 5 IMPLICATION FOR POLICIES

The paucity of reported data from LMIC makes assessing priority risk groups for vaccination and treatment difficult. Continued investment in capacity building for influenza surveillance is fundamental to understanding the role of influenza in association with serious outcomes; the need is universal, but urgent in LMIC. Although pregnant women, people with neurological conditions and people with immune‐suppressing conditions including HIV/AIDS are included in current recommendations for vaccination against influenza in HIC, governments and establishments such as the World Health Organization should ensure they are also considered as high priority groups for vaccination and treatment in LMIC.

## CONFLICT OF INTEREST

The authors declare that no conflict of interest exists.
