# Supplementary material for: Risk factors for serious outcomes associated with influenza illness in high‐ versus low‐ and middle‐income countries: Systematic literature review and meta‐analysis
Source: Influenza Other Respir Viruses. 2017 Dec 2;12(1):22–9. doi: 10.1111/irv.12504 (PMC5818335; doi:10.1111/irv.12504)
Supplement: Supplementary file 9 [file IRV-12-22-s009.doc]

**Supplementary material: Results of meta-analyses for *risk factors for severe outcomes associated with influenza illness: systematic literature review and meta-analysis,* Coleman et al. 2017*.***

Table 1a: Random effects model meta-analysis of risk factors associated with influenza illness: demographics

| **Outcome** | **Meas-ure** | **High Income Countries (HIC)** | | | | **Low and Middle Income Countries (LMIC)** | | | |
| --- | --- | --- | --- | --- | --- | --- | --- | --- | --- |
|  |  | **Estimate (95% CI)** | **I^2^** | **N** | **Citations** | **Estimate (95% CI)** | **I^2^** | **N** | **Citations** |
| **<5 years vs youths (5 to 19 years, or as per authors’ cut point of 24 years or younger)** | | | | | | | | | |
| Hospital admission | RR | 3.39 (3.00, 3.83) | 0 | 3 |  | 0.84 (0.62, 1.15) | -- | 1 | [^148^](#_ENREF_148) |
|  | OR | 2.75 (0.66, 11.3) | 93.6 | 2 |  | -- |  | 0 |  |
|  | pRR | 2.36 (1.42, 3.92) | 95.9 | 5 |  | -- |  |  |  |
| ICU  admission | RR | 0.61 (0.51, 0.74) | 43.8 | 8 |  | -- |  | 0 |  |
|  | OR | -- |  | 0 |  | -- |  | 0 |  |
|  | pRR | -- |  |  |  | -- |  |  |  |
| Critical (ICU, died) | RR | 0.90 (0.67, 1.20) | 0 | 2 |  | 1.90 (1.34, 2.69) | -- | 1 | [^118^](#_ENREF_118) |
|  | OR | -- |  | 0 |  | -- |  | 0 |  |
|  | pRR | -- |  |  |  | -- |  |  |  |
| Mortality  (all cause) | RR | 0.53 (0.18, 1.58) | 77.6 | 6 |  | 1.27 (0.86, 1.86) | 0 | 6 |  |
|  | OR | 1.07 (0.51, 2.26) | 0 | 2 |  | 1.33 (0.12, 14.8) | -- | 1 | [^14^](#_ENREF_14) |
|  | pRR | 0.68 (0.33, 1.39) | 70.0 | 8 |  | 1.27 (0.87, 1.85) | 0 | 7 |  |
| **<5 years vs younger adults (20-64 years, or as per author’s cut point of at least 16 years and, for younger adults, no older than 64 years)** | | | | | | | | | |
| Hospital admission | RR | 5.00 (4.28, 5.85) | -- | 1 | [^81^](#_ENREF_81) | 0.55 (0.40, 0.75) | -- | 1 | [^148^](#_ENREF_148) |
|  | OR | 13.7 (7.84, 24.1) | -- | 1 | [^70^](#_ENREF_70) | -- |  | 0 |  |
|  | pRR | 4.54 (3.59, 5.74) | 71.5 | 2 |  | -- |  |  |  |
| ICU  admission | RR | 0.46 (0.25, 0.83) | 96.4 | 6 |  | -- |  | 0 |  |
|  | OR | 0.28 (0.09, 0.84) | -- | 1 | [^70^](#_ENREF_70) | -- |  | 0 |  |
|  | pRR | 0.44 (0.25, 0.77) | 95.7 | 7 |  | -- |  |  |  |
| Critical (ICU, died) | RR | -- |  | 0 |  | 0.89 (0.74-1.08) | -- | 1 | [^118^](#_ENREF_118) |
|  | OR | 0.53 (0.16, 1.75) | -- | 1 | [^70^](#_ENREF_70) | -- |  | 0 |  |
|  | pRR | -- |  |  |  | -- |  |  |  |
| Mortality  (all cause) | RR | 0.11 (0.06, 0.20) | 45.9 | 7 |  | 0.30 (0.14, 0.63) | 51.7 | 5 |  |
|  | OR | 0.31 (0.18, 0.56) | 0 | 3 |  | 0.14 (0.03, 0.57) | -- | 1 | [^14^](#_ENREF_14) |
|  | pRR | 0.18 (0.08, 0.39) | 80.7 | 10 |  | 0.32 (0.17, 0.60) | 25.4 | 5 |  |

CI: confidence interval; N: number of studies; OR: odds ratio; RR: relative risk; pRR: Pooled RR for cohort and case-referent designs (zero cells given a value of 0.1)

Notes: Estimates are produced using random effects models: interpret estimates with caution when the number of studies is small and/or the I^2^ estimates are high. The number of citations will not equal N when >1 estimate provided (e.g., children & adults or pandemic & seasonal).

Table 1b: Random effects model meta-analysis of risk factors associated with influenza illness: demographics

| **Outcome** | **Meas-ure** | **High Income Countries (HIC)** | | | | **Low and Middle Income Countries (LMIC)** | | | |
| --- | --- | --- | --- | --- | --- | --- | --- | --- | --- |
|  |  | **Estimate (95% CI)** | **I^2^** | **N** | **Citations** | **Estimate (95% CI)** | **I^2^** | **N** | **Citations** |
| **<5 years vs older adults (65 years and older, or per authors’ cut-point of at least 55 years)** | | | | | | | | | |
| Hospital admission | RR | 5.66 (4.80, 6.67) | -- | 1 | [^81^](#_ENREF_81) | 0.36 (0.26, 0.50) | -- | 1 | [^148^](#_ENREF_148) |
|  | OR | 1.54 (0.72, 3.31) | -- | 1 | [^70^](#_ENREF_70) | -- |  | 0 |  |
|  | pRR | 2.53 (0.43, 14.9) | 99.4 | 2 |  | -- |  |  |  |
| ICU  admission | RR | 0.35 (0.22, 0.57) | 72.4 | 4 |  | -- |  | 0 |  |
|  | OR | 0.66 (0.15, 2.82) | -- | 1 | [^70^](#_ENREF_70) | -- |  | 0 |  |
|  | pRR | 0.37 (0.24, 0.58) | 66.6 | 5 |  | -- |  |  |  |
| Critical (ICU, died) | RR | -- |  | 0 |  | 0.57 (0.43-0.74) | -- | 1 | [^118^](#_ENREF_118) |
|  | OR | 0.22 (0.06, 0.76) | -- | 1 | [^70^](#_ENREF_70) | -- |  | 0 |  |
|  | pRR | -- |  |  |  | -- |  |  |  |
| Mortality  (all cause) | RR | 0.07 (0.04, 0.12) | 36.4 | 5 |  | 0.16 (0.10, 0.27) | 0 | 6 |  |
|  | OR | 0.17 (0.03, 0.99) | 51.0 | 3 |  | 0.08 (0.01, 0.44) | -- | 1 | [^14^](#_ENREF_14) |
|  | pRR | 0.12 (0.05, 0.29) | 77.6 | 8 |  | 0.15 (0.09, 0.25) | 0 | 7 |  |
| **Youths vs young adults (5-19 vs 20-64 years)** | | | | | | | | | |
| Hospital admission | RR | 1.44 (1.19, 1.73) | -- | 1 | [^81^](#_ENREF_81) | 0.65 (0.53, 0.80) | -- | 1 | [^148^](#_ENREF_148) |
|  | OR | -- |  | 0 |  | -- |  | 0 |  |
|  | pRR | -- |  |  |  | -- |  |  |  |
| ICU  admission | RR | 0.71 (0.38, 1.34) | 97.0 | 6 |  | -- |  | 0 |  |
|  | OR | -- |  | 0 |  | -- |  | 0 |  |
|  | pRR | -- |  |  |  | -- |  |  |  |
| Critical (ICU, died) | RR | -- |  | 0 |  | 0.59 (0.38, 0.93) | 76.4 | 2 |  |
|  | OR | -- |  | 0 |  | -- |  | 0 |  |
|  | pRR | -- |  |  |  | -- |  |  |  |
| Mortality  (all cause) | RR | 0.25 (0.08, 0.83) | 92.7 | 7 |  | 0.47 (0.30, 0.75) | 0 | 4 |  |
|  | OR | 0.30 (0.18, 0.50) | 0 | 2 |  | 0.10 (0.01, 0.76) | -- | 1 | [^14^](#_ENREF_14) |
|  | pRR | 0.30 (0.12, 0.74) | 92.4 | 9 |  | 0.31 (0.09, 0.99) | 19.6 | 5 |  |

CI: confidence interval; N: number of studies; OR: odds ratio; RR: relative risk; pRR: Pooled RR for cohort and case-referent designs (zero cells given a value of 0.1)

Notes: Estimates are produced using random effects models: interpret estimates with caution when the number of studies is small and/or the I^2^ estimates are high. The number of citations will not equal N when >1 estimate provided (e.g., children & adults or pandemic & seasonal).

Table 1c: Random effects model meta-analysis of risk factors associated with influenza illness: demographics

| **Outcome** | **Meas-ure** | **High Income Countries (HIC)** | | | | **Low and Middle Income Countries (LMIC)** | | | |
| --- | --- | --- | --- | --- | --- | --- | --- | --- | --- |
|  |  | **Estimate (95% CI)** | **I^2^** | **N** | **Citations** | **Estimate (95% CI)** | **I^2^** | **N** | **Citations** |
| **Youths vs older adults (15-19 years vs 65 years and older)** | | | | | | | | | |
| Hospital admission | RR | 1.62 (1.34, 1.97) | -- | 1 | [^81^](#_ENREF_81) | 0.42 (0.33, 0.54) | -- | 1 | [^148^](#_ENREF_148) |
|  | OR | -- |  | 0 |  | -- |  | 0 |  |
|  | pRR | -- |  |  |  | -- |  |  |  |
| ICU  admission | RR | 0.55 (0.39, 0.79) | 61.2 | 4 |  | -- |  | 0 |  |
|  | OR | -- |  | 0 |  | -- |  | 0 |  |
|  | pRR | -- |  |  |  | -- |  |  |  |
| Critical (ICU, died) | RR | -- |  | 0 |  | 0.43 (0.21, 0.89) | 89.3 | 2 |  |
|  | OR | -- |  | 0 |  | -- |  | 0 |  |
|  | pRR | -- |  |  |  | -- |  |  |  |
| Mortality  (all cause) | RR | 0.19 (0.07, 0.47) | 65.9 | 4 |  | 0.23 (0.02, 2.54) | 55.7 | 5 |  |
|  | OR | 0.19 (0.01, 2.83) | 84.5 | 2 |  | 0.06 (0.01, 0.55) | -- | 1 | [^14^](#_ENREF_14) |
|  | pRR | 0.23 (0.12, 0.45) | 71.2 | 6 |  | 0.18 (0.04, 0.89) | 45.1 | 6 |  |
| **Young vs older adults (20-64 years vs 65 years and older)** | | | | | | | | | |
| Hospital admission | RR | 0.23 (0.18, 0.68) | 98.0 | 5 |  | 0.65 (0.51, 0.84) | -- | 1 | [^148^](#_ENREF_148) |
|  | OR | 0.08 (0.04, 0.14) | -- | 1 | [^70^](#_ENREF_70) | -- |  | 0 |  |
|  | pRR | 0.23 (0.10, 0.53) | 97.6 | 6 |  | -- |  |  |  |
| ICU  admission | RR | 0.84 (0.54, 1.32) | 94.0 | 7 |  | -- |  | 0 |  |
|  | OR | 2.35 (0.77, 7.15) | -- | 1 | [^70^](#_ENREF_70) | -- |  | 0 |  |
|  | pRR | 0.91 (0.59, 1.40) | 93.2 | 8 |  | -- |  |  |  |
| Critical (ICU, died) | RR | 0.70 (0.48, 1.01) | 58.1 | 7 |  | 0.73 (0.58, 0.91) | 28.5 | 3 |  |
|  | OR | 1.07 (0.48, 2.42) | -- | 1 | [^70^](#_ENREF_70) | -- |  | 0 |  |
|  | pRR | 0.74 (0.52, 1.05) | 59.1 | 8 |  | -- |  |  |  |
| Mortality  (all cause) | RR | 0.53 (0.42, 0.70) | 61.7 | 12 |  | 0.52 (0.25, 1.05) | 62.3 | 7 |  |
|  | OR | 0.43 (0.13, 1.45) | 69.4 | 3 |  | 0.49 (0.16, 1.53) | 66.1 | 3 |  |
|  | pRR | 0.55 (0.45, 0.66) | 57.1 | 15 |  | 0.52 (0.30, 0.91) | 65.3 | 10 |  |

CI: confidence interval; N: number of studies; OR: odds ratio; RR: relative risk; pRR: Pooled RR for cohort and case-referent designs (zero cells given a value of 0.1)

Notes: Estimates are produced using random effects models: interpret estimates with caution when the number of studies is small and/or the I^2^ estimates are high. The number of citations will not equal N when >1 estimate provided (e.g., children & adults or pandemic & seasonal).

Table 1d: Random effects model meta-analysis of risk factors associated with influenza illness: demographics

| **Outcome** | **Meas-ure** | **High Income Countries (HIC)** | | | | **Low and Middle Income Countries (LMIC)** | | | |
| --- | --- | --- | --- | --- | --- | --- | --- | --- | --- |
|  |  | **Estimate (95% CI)** | **I^2^** | **N** | **Citations** | **Estimate (95% CI)** | **I^2^** | **N** | **Citations** |
| **Sex (Male vs. Female)** | | | | | | | | | |
| Hospital admission | RR | 1.18 (1.06, 1.30) | 66.0 | 16 |  | 1.25 (0.46, 3.37) | 79.8 | 2 |  |
|  | OR | 0.93 (0.71, 1.21) | 74.1 | 7 |  | 0.57 (0.42, 0.77) | -- | 1 | [^151^](#_ENREF_151) |
|  | pRR | 1.09 (1.01, 1.18) | 68.9 | 23 |  | 0.95 (0.60, 1.50) | 71.6 | 3 |  |
| ICU  admission | RR | 1.07 (0.98, 1.17) | 41.2 | 31 |  | 1.01 (0.95, 1.07) | 0 | 4 |  |
|  | OR | 0.55 (0.31, 0.97) | 0 | 2 |  | -- |  | 0 |  |
|  | pRR | 1.04 (0.95, 1.14) | 43.3 | 33 |  | -- |  |  |  |
| Critical (ICU, died) | RR | 1.15 (1.03 (1.27) | 39.2 | 14 |  | 0.97 (0.87, 1.07) | 41.9 | 3 |  |
|  | OR | 0.48 (0.27, 0.87) | -- | 1 | [^70^](#_ENREF_70) | -- |  | 0 |  |
|  | pRR | 1.13 (1.01, 1.27) | 50.6 | 15 |  | -- |  |  |  |
| Mortality  (all cause) | RR | 1.09 (0.99, 1.20) | 3.0 | 24 |  | 0.89 (0.78, 1.03) | 42.7 | 22 |  |
|  | OR | 0.84 (0.52, 1.36) | 45.2 | 3 |  | 1.26 (0.71, 2.21) | 61.0 | 5 |  |
|  | pRR | 1.07 (0.97, 1.17) | 9.3 | 27 |  | 0.92 (0.81, 1.05) | 45.2 | 27 |  |

CI: confidence interval; N: number of studies; OR: odds ratio; RR: relative risk; pRR: Pooled RR for cohort and case-referent designs (zero cells given a value of 0.1)

Notes: Estimates are produced using random effects models: interpret estimates with caution when the number of studies is small and/or the I^2^ estimates are high. The number of citations will not equal N when >1 estimate provided (e.g., children & adults or pandemic & seasonal). Publications do NOT include those that provided data by age and/or sex only.

Table 2a: Random effects model meta-analysis of risk factors associated with influenza illness: underlying conditions

| **Outcome** | **Meas-ure** | **High Income Countries (HIC)** | | | | **Low and Middle Income Countries (LMIC)** | | | |
| --- | --- | --- | --- | --- | --- | --- | --- | --- | --- |
|  |  | **Estimate (95% CI)** | **I^2^** | **N** | **Citations** | **Estimate (95% CI)** | **I^2^** | **N** | **Citations** |
| **Any chronic underlying condition (as defined by authors)** | | | | | | | | | |
| Hospital admission | RR | 2.41 (1.67, 3.47) | 96.9 | 11 |  | 2.50 (1.23, 5.06) | 84.5 | 3 |  |
|  | OR | 3.43 (2.28, 5.15) | 81.1 | 7 |  | -- |  | 0 |  |
|  | pRR | 2.22 (1.76, 2.80) | 95.3 | 18 |  | -- |  |  |  |
| ICU  admission | RR | 1.60 (1.23, 2.07) | 91.4 | 29 |  | 1.42 (0.86, 2.34) | 61.7 | 2 |  |
|  | OR | 1.20 (0.64, 2.27) | -- | 1 | [^70^](#_ENREF_70) | -- |  | 0 |  |
|  | pRR | 1.58 (1.22, 2.03) | 91.1 | 30 |  | -- |  |  |  |
| Critical (ICU, died) | RR | 1.29 (1.08, 1.55) | 72.2 | 13 |  | 2.07 (1.22, 3.52) | 97.3 | 7 |  |
|  | OR | 14.8 (5.23, 42.1) | -- | 1 | [^70^](#_ENREF_70) | -- |  | 0 |  |
|  | pRR | 1.38 (1.13, 1.68) | 77.5 | 14 |  | -- |  |  |  |
| Mortality  (all cause) | RR | 2.51 (1.66, 3.79) | 85.7 | 29 |  | 2.39 (1.66, 3.43) | 88.2 | 16 |  |
|  | OR | 4.57 (1.19, 17.6) | 90.2 | 3 |  | 3.30 (2.03, 5.36) | 36.4 | 4 |  |
|  | pRR | 2.56 (1.79, 3.66) | 84.5 | 35 |  | 2.37 (1.76, 3.18) | 85.5 | 20 |  |
| **Cardiac disease/condition (as defined by authors)** | | | | | | | | | |
| Hospital admission | RR | 2.24 (1.54, 3.27) | 89.4 | 13 |  | 2.18 (0.95, 5.01) | -- | 1 | [^165^](#_ENREF_165) |
|  | OR | 3.00 (1.36, 6.59) | 82.4 | 6 |  | Not estimable | -- | 1 | [^151^](#_ENREF_151) |
|  | pRR | 1.96 (1.58, 2.42) | 88.3 | 19 |  | 2.15 (1.98, 2.34) | 0 | 2 |  |
| ICU  admission | RR | 1.50 (1.15, 1.96) | 75.0 | 24 |  | 1.92 (1.60, 2.32) | 3.8 | 5 |  |
|  | OR | 1.50 (0.64, 3.48) | -- | 1 | [^70^](#_ENREF_70) | -- |  | 0 |  |
|  | pRR | 1.50 (1.16, 1.94) | 73.9 | 25 |  | -- |  |  |  |
| Critical (ICU, died) | RR | 1.62 (1.39, 1.90) | 58.8 | 19 |  | 2.94 (1.67, 5.18) | 97.6 | 4 |  |
|  | OR | 1.65 (0.77, 3.50) | -- | 1 | [^70^](#_ENREF_70) | -- |  | 0 |  |
|  | pRR | 1.62 (1.39, 1.88) | 56.5 | 20 |  | -- |  |  |  |
| Mortality  (all cause) | RR | 2.48 (1.75, 3.51) | 88.9 | 19 |  | 2.21 (1.59, 3.08) | 72.7 | 13 |  |
|  | OR | 4.77 (0.61, 37.3) | 86.8 | 4 |  | 4.70 (2.42, 9.12) | -- | 1 | [^153^](#_ENREF_153) |
|  | pRR | 2.50 (1.87, 3.36) | 88.6 | 23 |  | 2.18 (1.65, 2.90) | 71.7 | 14 |  |

CI: confidence interval; N: number of studies; OR: odds ratio; RR: relative risk; pRR: Pooled RR for cohort and case-referent designs (zero cells given a value of 0.1)

Notes: Estimates are produced using random effects models: interpret estimates with caution when the number of studies is small and/or the I^2^ estimates are high. The number of citations will not equal N when >1 estimate provided (e.g., children & adults or pandemic & seasonal).

Table 2b: Random effects model meta-analysis of risk factors associated with influenza illness: underlying conditions

| **Outcome** | **Meas-ure** | **High Income Countries (HIC)** | | | | **Low and Middle Income Countries (LMIC)** | | | |
| --- | --- | --- | --- | --- | --- | --- | --- | --- | --- |
|  |  | **Estimate (95% CI)** | **I^2^** | **N** | **Citations** | **Estimate (95% CI)** | **I^2^** | **N** | **Citations** |
| **Liver disease** | | | | | | | | | |
| Hospital admission | RR | 1.93 (0.89, 4.21) | 52.2 | 3 |  | -- |  | 0 |  |
|  | OR | 1.63 (0.76, 3.50) | 0 | 5 |  | Not estimable | -- | 1 | [^151^](#_ENREF_151) |
|  | pRR | 1.61 (1.07, 2.44) | 84.4 | 8 |  | 2.02 (1.81, 2.25) | -- | 1 |  |
| ICU  admission | RR | 2.08 (1.53, 2.83) | 68.9 | 10 |  | 1.49 (1.32, 1.69) | -- | 1 | [^193^](#_ENREF_193) |
|  | OR | 1.47 (0.16, 13.4) | -- | 1 | [^70^](#_ENREF_70) | -- |  | 0 |  |
|  | pRR | 2.06 (1.53, 2.78) | 65.8 | 11 |  | -- |  |  |  |
| Critical (ICU, died) | RR | 1.31 (1.08, 1.59) | 0 | 5 |  | 1.66 (0.87, 3.16) | 92.8 | 3 |  |
|  | OR | 1.04 (0.11, 9.52) | -- | 1 | [^70^](#_ENREF_70) | -- |  | 0 |  |
|  | pRR | 1.31 (1.08, 1.58) | 0 | 6 |  | -- |  |  |  |
| Mortality  (all cause) | RR | 6.92 (4.56, 10.5) | 13.5 | 6 |  | 1.46 (1.12, 1.89) | 0 | 4 |  |
|  | OR | Not estimable | -- | 1 | [^70^](#_ENREF_70) | 10.2 (1.19, 88.3) | -- | 1 | [^153^](#_ENREF_153) |
|  | pRR | 7.00 (4.73, 10.3) | 5.5 | 7 |  | 1.78 (1.23, 2.58) | 56.7 | 5 |  |
| **Neurological/neuromuscular condition (as defined by authors; if categorized separately, neurological included preferentially for these analyses)** | | | | | | | | | |
| Hospital admission | RR | 2.36 (1.74, 3.19) | 90.2 | 11 |  | -- |  | 0 |  |
|  | OR | 3.55 (2.18, 5.79) | 5.3 | 5 |  | -- |  | 0 |  |
|  | pRR | 2.09 (1.73, 2.54) | 86.6 | 16 |  | -- |  |  |  |
| ICU  admission | RR | 2.02 (1.50-2.73) | 86.7 | 25 |  | 2.72 (0.71, 10.4) | 98.0 | 2 |  |
|  | OR | 1.10 (0.31, 3.93) | -- | 1 | [^70^](#_ENREF_70) | -- |  | 0 |  |
|  | pRR | 1.98 (1.48-2.66) | 86.3 | 26 |  | -- |  |  | F: 1.50 (1.28-1.76) |
| Critical (ICU, died) | RR | 2.31 (1.66, 3.22) | 86.6 | 12 |  | 1.67 (0.91, 3.05) | 79.3 | 5 |  |
|  | OR | 1.12 (0.36, 3.51) | -- | 1 | [^70^](#_ENREF_70) | -- |  | 0 |  |
|  | pRR | 2.22 (1.61, 3.05) | 85.6 | 13 | F: 1.51 (1.36-1.68) | -- |  |  | F: 2.74 (2.44-3.06) |
| Mortality  (all cause) | RR | 3.61 (2.57, 5.06) | 63.8 | 17 |  | 2.52 (1.91, 3.32) | 4.2 | 8 |  |
|  | OR | 2.08 (0.69, 6.28) | 14.5 | 3 |  | 8.32 (3.06, 22.6) | -- | 1 | [^153^](#_ENREF_153) |
|  | pRR | 3.30 (2.39, 4.54) | 62.2 | 20 |  | 2.53 (2.13, 3.00) | 0 | 9 |  |

CI: confidence interval; N: number of studies; OR: odds ratio; RR: relative risk; pRR: Pooled RR for cohort and case-referent designs (zero cells given a value of 0.1)

Notes: Estimates are produced using random effects models: interpret estimates with caution when the number of studies is small and/or the I^2^ estimates are high. The number of citations will not equal N when >1 estimate provided (e.g., children & adults or pandemic & seasonal).

Table 2c: Random effects model meta-analysis of risk factors associated with influenza illness: underlying conditions

| **Outcome** | **Meas-ure** | **High Income Countries (HIC)** | | | | **Low and Middle Income Countries (LMIC)** | | | |
| --- | --- | --- | --- | --- | --- | --- | --- | --- | --- |
|  |  | **Estimate (95% CI)** | **I^2^** | **N** | **Citations** | **Estimate (95% CI)** | **I^2^** | **N** | **Citations** |
| **Diabetes mellitus (any type)** | | | | | | | | | |
| Hospital admission | RR | 2.51 (1.90, 3.32) | 76.3 | 13 |  | 4.27 (3.15, 5.78) | -- | 1 | [^165^](#_ENREF_165) |
|  | OR | 3.59 (1.91, 6.76) | 68.1 | 5 |  | Not estimable | -- | 1 | [^151^](#_ENREF_151) |
|  | pRR | 2.15 (1.78, 2.58) | 82.7 | 18 |  | 2.86 (1.32, 6.16) | 95.5 | 2 |  |
| ICU  admission | RR | 1.64 (1.28, 2.10) | 71.8 | 21 |  | 1.19 (0.53, 2.66) | 75.0 | 2 |  |
|  | OR | 1.58 (1.13, 2.21) | 0 | 3 |  | -- |  | 0 |  |
|  | pRR | 1.57 (1.28, 1.95) | 68.6 | 24 |  | -- |  |  |  |
| Critical (ICU, died) | RR | 1.52 (1.21, 1.90) | 63.3 | 14 |  | 2.10 (1.06, 4.16) | 93.1 | 5 |  |
|  | OR | 1.85 (0.86, 3.98) | -- | 1 | [^70^](#_ENREF_70) | -- |  | 0 |  |
|  | pRR | 1.53 (1.24, 1.88) | 60.4 | 15 |  | -- |  |  |  |
| Mortality  (all cause) | RR | 2.19 (1.41, 3.41) | 91.6 | 21 |  | 1.94 (1.45, 2.61) | 69.4 | 11 |  |
|  | OR | 3.28 (1.51, 7.16) | 70.0 | 4 |  | 5.02 (2.69, 9.36) | 40.8 | 2 |  |
|  | pRR | 2.15 (1.52, 3.03) | 90.1 | 25 |  | 2.08 (1.66, 2.60) | 66.9 | 13 |  |
| **Metabolic conditions/diseases (excluding diabetes mellitus if categorized separately by authors)** | | | | | | | | | |
| Hospital admission | RR | 0.36 (0.01, 156) | -- | 1 | [^93^](#_ENREF_93) | -- |  | 0 |  |
|  | OR | 4.97 (0.55, 44.7) | -- | 1 | [^70^](#_ENREF_70) | -- |  | 0 |  |
|  | pRR | 1.78 (1.14, 2.77) | 0 | 2 |  | -- |  |  |  |
| ICU  admission | RR | 1.43 (1.10, 1.87) | 0 | 3 |  | -- |  | 0 |  |
|  | OR | Not estimable | -- | 1 | [^70^](#_ENREF_70) | -- |  | 0 |  |
|  | pRR | 1.43 (1.10, 1.87) | 0 | 4 |  | -- |  |  |  |
| Critical (ICU, died) | RR | 1.38 (1.09, 1.74) | 62.9 | 5 |  | 1.73 (1.30, 2.30) | -- | 1 | [^191^](#_ENREF_191) |
|  | OR | Not estimable | -- | 1 | [^70^](#_ENREF_70) | -- |  | 0 |  |
|  | pRR | 1.37 (1.10, 1.71) | 54.8 | 6 |  | -- |  |  |  |
| Mortality  (all cause) | RR | 1.89 (1.22, 2.93) | 44.3 | 4 |  | 1.83 (1.00, 3.35) | 0 | 2 |  |
|  | OR | 1.47 (0.41, 5.26) | 0 | 3 |  | -- |  | 0 |  |
|  | pRR | 1.72 (1.30, 2.29) | 9.4 | 7 |  | -- |  |  |  |

CI: confidence interval; N: number of studies; OR: odds ratio; RR: relative risk; pRR: Pooled RR for cohort and case-referent designs (zero cells given a value of 0.1)

Notes: Estimates are produced using random effects models: interpret estimates with caution when the number of studies is small and/or the I^2^ estimates are high. The number of citations will not equal N when >1 estimate provided (e.g., children & adults or pandemic & seasonal).

Table 2d: Random effects model meta-analysis of risk factors associated with influenza illness: underlying conditions

| **Outcome** | **Meas-ure** | **High Income Countries (HIC)** | | | | **Low and Middle Income Countries (LMIC)** | | | |
| --- | --- | --- | --- | --- | --- | --- | --- | --- | --- |
|  |  | **Estimate (95% CI)** | **I^2^** | **N** | **Citations** | **Estimate (95% CI)** | **I^2^** | **N** | **Citations** |
| **Renal disease/condition** | | | | | | | | | |
| Hospital admission | RR | 2.55 (1.31, 4.97) | 94.8 | 10 |  | 1.74 (0.79, 3.86) | -- | 1 | [^165^](#_ENREF_165) |
|  | OR | 2.15 (1.20,3.87) | 46.9 | 6 |  | Not estimable | -- | 1 | [^151^](#_ENREF_151) |
|  | pRR | 2.00 (1.35, 2.99) | 93.7 | 16 |  | 2.01 (1.82, 2.21) | 0 | 2 |  |
| ICU  admission | RR | 1.35 (1.12, 1.64) | 0 | 14 |  | 1.57 (1.39, 1.79) | -- | 1 | [^193^](#_ENREF_193) |
|  | OR | 1.18 (0.33, 4.23) | -- | 1 | [^70^](#_ENREF_70) | -- |  | 0 |  |
|  | pRR | 1.35 (1.12, 1.63) | 0 | 15 |  | -- |  |  |  |
| Critical (ICU, died) | RR | 1.51 (1.24, 1.84) | 41.3 | 15 |  | 2.20 (1.38, 3.52) | 86.8 | 4 |  |
|  | OR | 0.83 (0.23, 2.95) | -- | 1 | [^70^](#_ENREF_70) | -- |  | 0 |  |
|  | pRR | 1.48 (1.22, 1.80) | 39.3 | 16 |  | -- |  |  |  |
| Mortality  (all cause) | RR | 2.26 (1.86, 2.75) | 0 | 11 |  | 2.09 (1.71, 2.56) | 0 | 8 |  |
|  | OR | 1.30 (0.42, 4.04) | 0 | 3 |  | 3.73 (1.23, 11.3) | -- | 1 | [^153^](#_ENREF_153) |
|  | pRR | 2.34 (1.71, 3.21) | 57.7 | 14 |  | 2.07 (1.72, 2.48) | 0 | 9 |  |

CI: confidence interval; N: number of studies; OR: odds ratio; RR: relative risk; pRR: Pooled RR for cohort and case-referent designs (zero cells given a value of 0.1)

Notes: Estimates are produced using random effects models: interpret estimates with caution when the number of studies is small and/or the I^2^ estimates are high. The number of citations will not equal N when >1 estimate provided (e.g., children & adults or pandemic & seasonal).

Table 2e: Random effects model meta-analysis of risk factors associated with influenza illness: underlying conditions

| **Outcome** | **Meas-ure** | **High Income Countries (HIC)** | | | | | **Low and Middle Income Countries (LMIC)** | | | |
| --- | --- | --- | --- | --- | --- | --- | --- | --- | --- | --- |
|  |  | **Estimate (95% CI)** | **I^2^** | **N** | **Citations** | **Estimate (95% CI)** | | **I^2^** | **N** | **Citations** |
| **Chronic lung disease (excluding asthma if categorized separately by authors)** | | | | | | | | | | |
| Hospital admission | RR | 2.17 (1.67, 2.81) | 83.8 | 13 |  | 2.22 (1.33, 3.72) | | -- | 1 | [^165^](#_ENREF_165) |
|  | OR | 3.82 (2.10, 6.93) | 69.5 | 6 |  | Not estimable | | -- | 1 | [^151^](#_ENREF_151) |
|  | pRR | 1.96 (1.69, 2.27) | 81.3 | 19 |  | 2.03 (1.84, 2.25) | | 0 | 2 |  |
| ICU  admission | RR | 1.52 (1.22, 1.89) | 68.0 | 27 |  | 1.64 (1.50, 1.79) | | 0 | 3 |  |
|  | OR | 1.67 (0.74, 3.76) | -- | 1 | [^70^](#_ENREF_70) | -- | |  | 0 |  |
|  | pRR | 1.52 (1.23, 1.88) | 66.8 | 28 |  | -- | |  |  |  |
| Critical (ICU, died) | RR | 1.59 (1.34, 1.89) | 76.4 | 2 |  | 1.82 (1.27, 2.62) | | 91.9 | 6 |  |
|  | OR | 1.76 (0.85, 3.67) | -- | 1 | [^70^](#_ENREF_70) | -- | |  | 0 |  |
|  | pRR | 1.59 (1.34, 1.87) | 75.3 | 21 |  | -- | |  |  |  |
| Mortality  (all cause) | RR | 1.44 (1.07, 1.95) | 81.1 | 17 |  | 1.28 (0.94, 1.74) | | 65.0 | 15 |  |
|  | OR | 3.83 (0.71, 20.7) | 85.8 | 4 |  | 1.84 (0.92, 3.68) | | 0 | 2 |  |
|  | pRR | 1.51 (1.12, 2.05) | 87.7 | 21 |  | 1.29 (0.98, 1.69) | | 60.9 | 17 |  |
| **Asthma** | | | | | | | | | | |
| Hospital admission | RR | 1.62 (0.64, 4.11) | 97.7 | 7 |  | -- | |  | 0 |  |
|  | OR | 1.98 (1.48, 2.65) | 20.4 | 4 |  | -- | |  | 0 |  |
|  | pRR | 1.56 (1.02, 2.40) | 96.2 | 11 |  | -- | |  |  |  |
| ICU  admission | RR | 0.91 (0.72, 1.14) | 54.8 | 24 |  | 1.30 (1.10, 1.53) | | 0 | 5 |  |
|  | OR | 0.89 (0.42, 1.92) | 18.2 | 3 |  | -- | |  | 0 |  |
|  | pRR | 0.91 (0.73, 1.12) | 50.8 | 27 |  | -- | |  |  |  |
| Critical (ICU, died) | RR | 0.80 (0.62, 1.02) | 71.4 | 9 |  | 1.17 (0.49, 2.81) | | 88.7 | 3 |  |
|  | OR | 0.73 (0.35, 1.53) | -- | 1 | [^70^](#_ENREF_70) | -- | |  | 0 |  |
|  | pRR | 0.79 (0.63, 1.00) | 67.9 | 10 |  | -- | |  |  |  |
| Mortality  (all cause) | RR | 0.48 (0.33, 0.71) | 47.3 | 12 |  | 0.67 (0.40, 1.12) | | 47.6 | 6 |  |
|  | OR | 1.19 (0.33, 4.28) | 68.9 | 4 |  | 0.68 (0.36, 1.28) | | -- | 1 | [^153^](#_ENREF_153) |
|  | pRR | 0.60 (0.35, 1.03) | 84.1 | 16 |  | 0.68 (0.45, 1.01) | | 42.3 | 7 |  |

CI: confidence interval; N: number of studies; OR: odds ratio; RR: relative risk; pRR: Pooled RR for cohort and case-referent designs (zero cells given a value of 0.1)

Notes: Estimates are produced using random effects models: interpret estimates with caution when the number of studies is small and/or the I^2^ estimates are high. The number of citations will not equal N when >1 estimate provided (e.g., children & adults or pandemic & seasonal).

Table 2f: Random effects model meta-analysis of risk factors associated with influenza illness: underlying conditions

| **Outcome** | **Meas-ure** | **High Income Countries (HIC)** | | | | **Low and Middle Income Countries (LMIC)** | | | |
| --- | --- | --- | --- | --- | --- | --- | --- | --- | --- |
|  |  | **Estimate (95% CI)** | **I^2^** | **N** | **Citations** | **Estimate (95% CI)** | **I^2^** | **N** | **Citations** |
| **Tobacco smoking** | | | | | | | | | |
| Hospital admission | RR | 1.41 (1.03, 1.94) | 60.2 | 5 |  | -- |  | 0 |  |
|  | OR | 1.35 (1.00, 1.83) | 48.7 | 5 |  | -- |  | 0 |  |
|  | pRR | 1.24 (1.07, 1.43) | 61.5 | 10 |  | -- |  |  |  |
| ICU  admission | RR | 1.53 (1.02, 2.29) | 78.6 | 8 |  | 1.39 (0.66, 2.93) | -- | 1 | [^178^](#_ENREF_178) |
|  | OR | 1.43 (0.64, 3.18) | -- | 1 | [^70^](#_ENREF_70) | -- |  | 0 |  |
|  | pRR | 1.51 (1.05, 2.17) | .754 | 9 |  | -- |  |  |  |
| Critical (ICU, died) | RR | 1.27 (1.05, 1.55) | 35.8 | 7 |  | -- |  | 0 |  |
|  | OR | 1.29 (0.62, 2.71) | -- | 1 | [^70^](#_ENREF_70) | -- |  | 0 |  |
|  | pRR | 1.25 (1.06, 1.49) | 25.2 | 8 |  | -- |  |  |  |
| Mortality  (all cause) | RR | 1.43 (0.36, 5.67) | 77.5 | 3 |  | 1.66 (1.30, 2.12) | 17.7 | 5 |  |
|  | OR | 2.34 (0.46, 12.0) | 74.9 | 2 |  | 1.24 (0.84, 1.83) | 0 | 5 |  |
|  | pRR | 1.50 (0.75, 3.02) | 68.4 | 5 |  | 1.44 (1.17, 1.78) | 26.5 | 10 |  |
| **Hematologic condition** | | | | | | | | | |
| Hospital admission | RR | 1.34 (1.10, 1.62) | 0 | 2 |  | -- |  | 0 |  |
|  | OR | 5.80 (0.89, 37.6) | 14.3 | 3 |  | -- |  | 0 |  |
|  | pRR | 1.85 (1.16, 2.94) | 94.3 | 5 |  | -- |  |  |  |
| ICU  admission | RR | 0.81 (0.58, 1.13) | 0 | 11 |  | 4.73 (2.86, 7.84) | -- | 1 | [^196^](#_ENREF_196) |
|  | OR | 2.01 (0.52, 7.71) | -- | 1 | [^70^](#_ENREF_70) | -- |  | 0 |  |
|  | pRR | 0.88 (0.64, 1.20) | 0 | 12 |  | -- |  |  |  |
| Critical (ICU, died) | RR | 0.59 (0.10, 3.59) | 73.6 | 2 |  | 0.23 (0.03, 1.65) | -- | 1 | [^16^](#_ENREF_16) |
|  | OR | 1.41 (0.37, 5.38) | -- | 1 | [^70^](#_ENREF_70) | -- |  | 0 |  |
|  | pRR | 0.97 (0.47, 1.98) | 47.9 | 3 |  | -- |  |  |  |
| Mortality  (all cause) | RR | 3.00 (2.32, 3.87) | 19.7 | 2 |  | 2.06 (1.44, 2.93) | 0 | 3 |  |
|  | OR | Not estimable | -- | 1 | [^70^](#_ENREF_70) | -- |  | 0 |  |
|  | pRR | 3.05 (2.47, 3.76) | 0 | 3 |  | -- |  |  |  |

CI: confidence interval; N: number of studies; OR: odds ratio; RR: relative risk; pRR: Pooled RR for cohort and case-referent designs (zero cells given a value of 0.1)

Notes: Estimates are produced using random effects models: interpret estimates with caution when the number of studies is small and/or the I^2^ estimates are high. The number of citations will not equal N when >1 estimate provided (e.g., children & adults or pandemic & seasonal).

Table 2g: Random effects model meta-analysis of risk factors associated with influenza illness: underlying conditions

| **Outcome** | **Meas-ure** | **High Income Countries (HIC)** | | | | | **Low and Middle Income Countries (LMIC)** | | | |
| --- | --- | --- | --- | --- | --- | --- | --- | --- | --- | --- |
|  |  | **Estimate (95% CI)** | **I^2^** | **N** | **Citations** | **Estimate (95% CI)** | | **I^2^** | **N** | **Citations** |
| **Immune suppressing condition** | | | | | | | | | | |
| Hospital admission | RR | 2.09 (1.22, 3.59) | 92.6 | 11 |  | -- | |  | 0 |  |
|  | OR | 2.35 (1.33, 4.15) | 29.8 | 3 |  | Not estimable | |  | 1 | [^151^](#_ENREF_151) |
|  | pRR | 2.01 (1.32, 3.04) | 92.0 | 14 |  | 2.00 (1.81, 2.22) | | -- | 1 |  |
| ICU  admission | RR | 0.90 (0.71, 1.15) | 43.9 | 22 |  | 1.38 (1.17, 1.63) | | 0 | 2 |  |
|  | OR | 1.07 (0.35, 3.24) | -- | 1 | [^70^](#_ENREF_70) | -- | |  | 0 |  |
|  | pRR | 0.91 (0.72, 1.14) | 41.3 | 23 |  | -- | |  |  |  |
| Critical (ICU, died) | RR | 1.36 (1.14, 1.63) | 49.5 | 14 |  | 1.89 (0.98, 3.67) | | 92.3 | 4 |  |
|  | OR | 2.42 (1.02, 5.72) | -- | 1 | [^70^](#_ENREF_70) | -- | |  | 0 |  |
|  | pRR | 1.39 (1.17, 1.65) | 49.2 | 15 |  | -- | |  |  |  |
| Mortality  (all cause) | RR | 2.58 (1.63, 4.07) | 88.3 | 19 |  | 2.73 (2.18, 3.43) | | 29.8 | 10 |  |
|  | OR | 3.47 (0.67, 18.0) | 72.2 | 3 |  | 2.36 (0.87, 6.41) | | 64.8 | 2 |  |
|  | pRR | 2.60 (1.70, 3.97) | 87.6 | 22 |  | 2.46 (1.99, 3.04) | | 42.5 | 12 |  |
| **Cancer/malignancy** | | | | | | | | | | |
| Hospital admission | RR | 2.17 (1.63, 2.91) | 48.1 | 6 |  | -- | |  | 0 |  |
|  | OR | 2.64 (0.52, 13.4) | 55.9 | 3 |  | Not estimable | |  | 1 | [^151^](#_ENREF_151) |
|  | pRR | 2.03 (1.53, 2.68) | 59.8 | 9 |  | 1.98 (1.75, 2.25) | | -- | 1 |  |
| ICU  admission | RR | 1.12 (0.92, 1.36) | 0 | 12 |  | 1.54 (0.21, 11.10) | | -- | 1 | [^26^](#_ENREF_26) |
|  | OR | 1.88 (0.34, 10.2) | 27.7 | 3 |  | -- | |  | 0 |  |
|  | pRR | 1.16 (0.96, 1.40) | 0 | 15 |  | -- | |  |  |  |
| Critical (ICU, died) | RR | 1.32 (1.00, 1.74) | 24.1 | 6 |  | 1.25 (0.40, 3.83) | | 37.3 | 2 |  |
|  | OR | 6.53 (1.07, 40.0) | -- | 1 | [^70^](#_ENREF_70) | -- | |  | 0 |  |
|  | pRR | 1.56 (1.08, 2.24) | 53.6 | 7 |  | -- | |  |  |  |
| Mortality  (all cause) | RR | 3.85 (2.52, 5.89) | 74.8 | 9 |  | 6.21 (3.39, 11.36) | | 0 | 4 |  |
|  | OR | 12.5 (1.59, 98.7) | 67.5 | 2 |  | -- | |  | 0 |  |
|  | pRR | 4.36 (2.87, 6.64) | 76.1 | 11 |  |  | |  |  |  |

CI: confidence interval; N: number of studies; OR: odds ratio; RR: relative risk; pRR: Pooled RR for cohort and case-referent designs (zero cells given a value of 0.1)

Notes: Estimates are produced using random effects models: interpret estimates with caution when the number of studies is small and/or the I^2^ estimates are high. The number of citations will not equal N when >1 estimate provided (e.g., children & adults or pandemic & seasonal).

Table 2h: Random effects model meta-analysis of risk factors associated with influenza illness: underlying conditions (pregnancy)

| **Outcome** | **Meas-ure** | **High Income Countries (HIC)** | | | | **Low and Middle Income Countries (LMIC)** | | | |
| --- | --- | --- | --- | --- | --- | --- | --- | --- | --- |
|  |  | **Estimate** | **I^2^** | **N** | **Citations** | **Estimate** | **I^2^** | **N** | **Citations** |
| **Pregnancy (including pregnant and early postpartum)** | | | | | | | | | |
| Hospital admission | RR | 1.53 (0.75, 3.13) | 96.9 | 6 |  | 3.58 (1.91, 6.68) | -- | 1 | [^165^](#_ENREF_165) |
|  | OR | 0.97 (0.67, 1.42) | 0 | 2 |  | 10.03 (3.59, 28.0) | -- | 1 | [^151^](#_ENREF_151) |
|  | pRR | 1.41 (0.88, 2.26) | 95.7 | 8 |  | 2.20 (1.04, 5.07) | 89.4 | 2 |  |
| ICU  admission | RR | 0.95 (0.68, 1.33) | 78.0 | 20 |  | 0.98 (0.75,1.28) | 9.4 | 4 |  |
|  | OR | 1.99 (0.25, 15.6) | 56.8 | 2 |  | -- |  | 0 |  |
|  | pRR | 0.98 (0.71, 1.36) | 78.0 | 22 |  | -- |  |  |  |
| Critical (ICU, died) | RR | 0.62 (0.38, 1.03) | 66.1 | 10 |  | 1.52 (0.91, 2.52) | 92.6 | 7 |  |
|  | OR | 0.45 (0.06, 3.66) | -- | 1 | [^70^](#_ENREF_70) | -- |  | 0 |  |
|  | pRR | 0.62 (0.38, 1.00) | 62.6 | 11 |  | -- |  |  |  |
| Mortality  (all cause) | RR | 0.45 (0.27, 0.76) | 25.2 | 19 |  | 1.50 (0.89, 2.51) | 92.3 | 20 |  |
|  | OR | 0.43 (0.11, 1.69) | 0 | 2 |  | 2.64 (0.41, 16.8) | 88.4 | 4 |  |
|  | pRR | 0.46 (0.29, 0.71) | 18.5 | 21 |  | 1.59 (1.01, 2.54) | 91.9 | 24 |  |

CI: confidence interval; N: number of studies; OR: odds ratio; RR: relative risk; pRR: Pooled RR for cohort and case-referent designs (zero cells given a value of 0.1)

Notes: Estimates are produced using random effects models: interpret estimates with caution when the number of studies is small and/or the I^2^ estimates are high. The number of citations will not equal N when >1 estimate provided (e.g., children & adults or pandemic & seasonal).

Table 2i: Meta-analysis of risk factors associated with influenza illness: underlying conditions

| **Outcome** | **Meas-ure** | **High Income Countries (HIC)** | | | | **Low and Middle Income Countries (LMIC)** | | | |
| --- | --- | --- | --- | --- | --- | --- | --- | --- | --- |
|  |  | **Estimate (95% CI)** | **I^2^** | **N** | **Citations** | **Estimate (95% CI)** | **I^2^** | **N** | **Citations** |
| **Human immunodeficiency virus (HIV) infection** | | | | | | | | | |
| Hospital admission | RR | -- |  | 0 |  | -- |  | 0 |  |
|  | OR | 0.82 (0.20, 3.29) | 78.5 | 3 |  | -- |  | 0 |  |
|  | pRR | 0.83 (0.33, 2.10) | 87.4 | 3 |  | -- |  |  |  |
| ICU  admission | RR | 1.09 (0.83, 1.44) | 7.6 | 2 |  | -- |  | 0 |  |
|  | OR | -- |  | 0 |  | -- |  | 0 |  |
|  | pRR | -- |  |  |  | -- |  |  |  |
| Critical (ICU, died) | RR | 1.59 (0.59, 4.26) | 57.5 | 2 |  | -- |  | 0 |  |
|  | OR | -- |  | 0 |  | -- |  | 0 |  |
|  | pRR | -- |  |  |  | -- |  |  |  |
| Mortality  (all cause) | RR | 0.96 (0.38, 2.44) | -- | 1 | [^141^](#_ENREF_141) | 2.19 (1.29, 3.71) | 0 | 3 |  |
|  | OR | Not estimable | -- | 2 |  | -- |  | 0 |  |
|  | pRR | 0.94 (0.37, 2.38) | 0 | 3 |  | -- |  |  |  |
| **Tuberculosis (TB)** | | | | | | | | | |
| Hospital admission | RR | -- |  | 0 |  | -- |  | 0 |  |
|  | OR | -- |  | 0 |  | -- |  | 0 |  |
|  | pRR | -- |  |  |  | -- |  |  |  |
| ICU  admission | RR | -- |  | 0 |  | -- |  | 0 |  |
|  | OR | -- |  | 0 |  | -- |  | 0 |  |
|  | pRR | -- |  |  |  | -- |  |  |  |
| Critical (ICU, died) | RR | -- |  | 0 |  | 1.67 (0.75, 3.73) | -- | 1 | [^118^](#_ENREF_118) |
|  | OR | -- |  | 0 |  | -- |  | 0 |  |
|  | pRR | -- |  |  |  | -- |  |  |  |
| Mortality  (all cause) | RR | 3.59 (1.17, 11.0) | -- | 1 | [^186^](#_ENREF_186) | 4.40 (2.51, 7.73) | 0 | 3 |  |
|  | OR | -- |  | 0 |  | -- |  | 0 |  |
|  | pRR | -- |  |  |  | -- |  |  |  |

CI: confidence interval; N: number of studies; OR: odds ratio; RR: relative risk; pRR: Pooled RR for cohort and case-referent designs (zero cells given a value of 0.1)

Notes: Estimates are produced using random effects models: interpret estimates with caution when the number of studies is small and/or the I^2^ estimates are high. The number of citations will not equal N when >1 estimate provided (e.g., children & adults or pandemic & seasonal).

Table 2j: Random effects model meta-analysis of risk factors associated with influenza illness: underlying conditions (obesity)

| **Outcome** | **Meas-ure** | **High Income Countries (HIC)** | | | | | **Low and Middle Income Countries (LMIC)** | | | |
| --- | --- | --- | --- | --- | --- | --- | --- | --- | --- | --- |
|  |  | **Estimate (95% CI)** | **I^2^** | **N** | **Citations** | **Estimate (95% CI)** | | **I^2^** | **N** | **Citations** |
| **Morbid obesity (≥40 kg/m^2^)** | | | | | | | | | | |
| Hospital admission | RR | 1.34 (1.07, 1.69) | 0 | 3 |  | -- | |  | 0 |  |
|  | OR | 5.41 (2.64, 11.1) | 0 | 2 |  | -- | |  | 0 |  |
|  | pRR | 1.65 (1.36, 5.01) | 44.8 | 5 |  | -- | |  |  |  |
| ICU  admission | RR | 2.76 (1.41, 5.42) | 89.6 | 3 |  | 1.51 (1.03-2.19) | | -- | 1 | [^193^](#_ENREF_193) |
|  | OR | 0.97 (0.21, 4.48) | -- | 1 | [^70^](#_ENREF_70) | -- | |  | 0 |  |
|  | pRR | 2.38 (1.28, 4.42) | 85.4 | 4 |  | -- | |  |  |  |
| Critical (ICU, died) | RR | 1.38 (1.23, 1.54) | 0 | 5 |  | 2.82 (1.76, 4.51) | | 28.8 | 2 |  |
|  | OR | 1.72 (0.52, 5.67) | -- | 1 | [^70^](#_ENREF_70) | -- | |  | 0 |  |
|  | pRR | 1.38 (1.24, 1.55) | 0 | 6 |  | -- | |  |  |  |
| Mortality  (all cause) | RR | 0.88 (0.36, 2.17) | 88.5 | 4 |  | 3.66 (0.09, 142) | | 94.4 | 2 |  |
|  | OR | 1.61 (0.20, 13.2) | -- | 1 | [^70^](#_ENREF_70) | 7.86 (4.56, 13.6) | | -- | 1 | [^153^](#_ENREF_153) |
|  | pRR | 0.94 (0.41, 2.15) | 84.8 | 5 |  | 3.40 (0.87, 13.3) | | 86.4 | 3 |  |
| **Obesity (≥30 kg/m^2^)** | | | | | | | | | | |
| Hospital admission | RR | 1.71 (1.37, 2.13) | 75.9 | 12 |  | 2.96 (1.60, 5.45) | | -- | 1 | [^165^](#_ENREF_165) |
|  | OR | 6.21 (2.14, 18.0) | -- | 1 | [^73^](#_ENREF_73) | Not estimable | | -- | 1 | [^151^](#_ENREF_151) |
|  | pRR | 1.71 (1.41, 2.07) | 75.6 | 13 |  | 2.47 (2.16, 2.82) | | 0 | 2 |  |
| ICU  admission | RR | 1.79 (1.19, 2.70) | 86.6 | 18 |  | 2.61 (0.54-12.5) | | 97.9 | 3 |  |
|  | OR | 1.05 (0.57, 1.93) | 0 | 3 |  | -- | |  | 0 |  |
|  | pRR | 1.64 (1.12, 2.39) | 85.1 | 21 |  | -- | |  |  |  |
| Critical (ICU, died) | RR | 1.70 (1.31, 2.21) | 68.9 | 11 |  | 1.64 (0.97, 2.78) | | 94.4 | 4 |  |
|  | OR | 1.38 (0.80, 2.39) | 0 | 2 | [^70^](#_ENREF_70) | -- | |  | 0 |  |
|  | pRR | 1.62 (1.29, 2.04) | 63.3 | 13 |  | -- | |  |  |  |
| Mortality  (all cause) | RR | 1.72 (0.92, 3.21) | 95.9 | 19 |  | 2.05 (1.28, 3.28) | | 88.9 | 10 |  |
|  | OR | 1.70 (0.20, 14.4) | 94.8 | 4 |  | 2.69 (0.97, 7.48) | | 75.9 | 4 |  |
|  | pRR | 1.64 (0.97, 2.78) | 95.9 | 23 |  | 1.95 (1.35, 2.82) | | 88.6 | 14 |  |

CI: confidence interval; N: number of studies; OR: odds ratio; RR: relative risk; pRR: Pooled RR for cohort and case-referent designs (zero cells given a value of 0.1)

Notes: Estimates are produced using random effects models: interpret estimates with caution when the number of studies is small and/or the I^2^ estimates are high. The number of citations will not equal N when >1 estimate provided (e.g., children & adults or pandemic & seasonal).

**Bibliography**

**1.** Abdelaty NM. Risk factors and prognostic criteria in 230 patients with influenza A (H1N1) infection. *Egypt J Chest Dis Tuberc* 2013;62:1-8.

**2.** Adeniji KA, Cusack R. The Simple Triage Scoring System (STSS) successfully predicts mortality and critical care resource utilization in H1N1 pandemic flu: a retrospective analysis. *Crit Care* 2011;15:R39.

**3.** Adlhoch C, Wadl M, Behnke M, Pena Diaz LA, Clausmeyer J, Eckmanns T. Pandemic influenza A(H1)pdm09 in hospitals and intensive care units - results from a new hospital surveillance, Germany 2009/2010. *Influenza Other Respir Viruses* 2012;6:e162-e168.

**4.** Al-Awaidy S, Hamid S, Al Obaidani I, et al. The burden of influenza-associated hospitalizations in Oman, January 2008-June 2013. *PLoS ONE* 2015;10 (12).

**5.** Allam RR, Murhekar MV, Tadi GP, Udaragudi PR. Descriptive epidemiology of novel influenza A (H1N1), Andhra Pradesh 2009-2010. *Indian J Public Health* 2013;57:161-165.

**6.** Allard R, Leclerc P, Tremblay C, Tannenbaum TN. Diabetes and the severity of pandemic influenza A (H1N1) infection. *Diabetes Care* 2010;33:1491-1493.

**7.** Amaravathi KS, Sakuntala P, Sudarsi B, Manohar S, Nagamani R, Rao SR. Clinical profile and outcome of recent outbreak of influenza A H1N1 (swine flu) at a tertiary care center in Hyderabad, Telangana. *Ann Trop Med Public Health* 2015;8:267-271.

**8.** Angelo SJ, Marshall PS, Chrissoheris MP, Chaves AM. Clinical characteristics associated with poor outcome in patients acutely infected with influenza A. *Connecticut Medicine* 2004;68:199-205.

**9.** Archer B, Cohen C, Naidoo D, et al. Interim report on pandemic H1N1 influenza virus infections in South Africa, April to October 2009: epidemiology and factors associated with fatal cases. *Euro Surveillance* 2009;14.

**10.** Armstrong M, Fica A, Dabanch J, Olivares F, Fasce R, Triantafilo V. Morbidity and mortality associated to influenza A (H1N1) 2009 admissions in two hospitals of the Metropolitan area and analysis of its economic impact. *Revista Chilena de Infectologia* 2012;29:664-671.

**11.** Ayatollahi J, Golestan M, Sharif MR, Esform E, Shahcheraghi SH. Investigation of the relationship between demographic characteristics and frequency of mortality in certain cases of influenza A (H1N1) from Yazd province (Iran). *Jundishapur J Microbiol* 2013;6 (10).

**12.** Azziz-Baumgartner E, Cabrera AM, Chang L, et al. Mortality, severe acute respiratory infection, and influenza-like illness associated with influenza A(H1N1)pdm09 in Argentina, 2009. *PLoS ONE* 2012;7 (10).

**13.** Bagdure D, Curtis DJ, Dobyns E, Glode MP, Dominguez SR. Hospitalized children with 2009 pandemic influenza A (H1N1): comparison to seasonal influenza and risk factors for admission to the ICU. *PLoS ONE* 2010;5:e15173.

**14.** Balaganesakumar SR, Murhekar MV, Swamy KK, Kumar MR, Manickam P, Pandian PRT. Risk factors associated with death among influenza A (H1N1) patients, Tamil Nadu, India, 2010. *J Postgrad Med* 2013;59:9-14.

**15.** Barakat A, Ihazmad H, El Falaki F, Tempia S, Cherkaoui I, El Aouad R. 2009 pandemic influenza a virus subtype H1N1 in Morocco, 2009-2010: epidemiology, transmissibility, and factors associated with fatal cases. *J Infect Dis* 2012;206:S94-S100.

**16.** Barrau M, Larrieu S, Cassadou S, et al. Hospitalized cases of influenza A(H1N1)pdm09 in the French territories of the Americas, July 2009-March 2010. *Pan American Journal of Public Health* 2012;32:124-130.

**17.** Bassetti M, Parisini A, Calzi A, et al. Risk factors for severe complications of the novel influenza A (H1N1): Analysis of patients hospitalized in Italy. *Clinical Microbiology & Infection* 2011;17:247-250.

**18.** Bauernfeind S, Bruennler T, Ehrenstein B, et al. Pandemic and post-pandemic influenza A (H1N1) seasons in a tertiary care university hospital - High rate of complications compared to previous influenza seasons. *Infection* 2013;41:145-150.

**19.** Berczy J, Sabel KG, Lundstrom R, Stefansson M. Hong Kong influenza in a group of patients with a high mortality. *Lakartidningen* 1971;68:722-726.

**20.** Bettinger JA, Sauve LJ, Scheifele DW, et al. Pandemic influenza in Canadian children: A summary of hospitalized pediatric cases. *Vaccine* 2010;28:3180-3184.

**21.** Brandsaeter BJ, Pillgram M, Berild D, Kjekshus H, Kran AMB, Bergersen BM. Hospitalised patients with suspected 2009 H1N1 Influenza A in a hospital in Norway, July - December 2009. *BMC Infect Dis* 2011;11.

**22.** Buda S, Kopke K, Haas W. Epidemiological characteristics of the influenza pandemic (H1N1) 2009 in Germany based on the mandatory notification of cases. *Bundesgesundheitsblatt Gesundheitsforschung Gesundheitsschutz* 2010;53:1223-1230.

**23.** Burton C, Moore D, Bettinger JA, et al. Burden of seasonal influenza in children with neurodevelopmental conditions. *Pediatr Infect Dis J* 2014;33:710-714.

**24.** Camargo LFA, De Sandes-Freitas TV, Silva CDR, et al. Morbimortality of pandemic influenza a H1N1 infection in kidney transplant recipients requiring hospitalization: A comparative analysis with nonimmunocompromised patients. *Transplantation* 2012;93:69-72.

**25.** Campbell A, Rodin R, Kropp R, et al. Risk of severe outcomes among patients admitted to hospital with pandemic (H1N1) influenza. *Canadian Medical Association Journal* 2010;182:349-355.

**26.** Canak G, Kovacevic N, Vukadinov J, et al. Clinical features, treatments and outcomes of influenza A (H1N1) 2009 among the hospitalized patients in the clinic for infectious diseases in Novi Sad. *Vojnosanitetski Pregled* 2013;70:155-162.

**27.** Capelastegui A, Quintana JM, Bilbao A, et al. Score to identify the severity of adult patients with influenza A (H1N1) 2009 virus infection at hospital admission. *European Journal of Clinical Microbiology & Infectious Diseases* 2012;31:2693-2701.

**28.** Chaves SS, Aragon D, Bennett N, et al. Patients hospitalized with laboratory-confirmed influenza during the 2010-2011 influenza season: Exploring disease severity by virus type and subtype. *J Infect Dis* 2013;208:1305-1314.

**29.** Chaves SS, Perez A, Farley MM, et al. The burden of influenza hospitalizations in infants from 2003 to 2012, United States. *Pediatr Infect Dis J* 2014;33:912-919.

**30.** Chawla R, Kansal S, Chauhan M, Jain A, Jibhkate B. Predictors of mortality and length of stay in hospitalized cases of 2009 influenza A (H1N1): Experiences of a tertiary care center. *Indian J Crit Care Med* 2013;17:275-282.

**31.** Chen Y, Xu X, Li S, Feng X, Li S, Zhao L. Clinical analysis of severe novel influenza A (H1N1) virus infection in pregnant women: a report of 13 cases. *Chinese Journal of Practical Internal Medicine* 2010;30:10-12.

**32.** Cheng AC, Holmes M, Senanayake S, et al. Influenza epidemiology in adults admitted to sentinel Australian hospitals in 2014: the influenza complications alert network (FLUCAN). *Commun Dis Intell* 2015;39:E355-E360.

**33.** Cheraghi Z, Irani AD, Rezaiean S, et al. Influenza A (H1N1) in Hamedan Province, Western Iran in 2009: A case-control study. *J Res Health Sci* 2010;10:15-21.

**34.** Chippirraz EL, Sorli L, Montero M, et al. Predictive factors for pneumonia in adults infected with the new pandemic A (H1H1) influenza virus. *Revista Espanola De Quimioterapia* 2011;24:204-208.

**35.** Chowell G, Ayala A, Berisha V, Viboud C, Schumacher M. Risk factors for mortality among 2009 A/H1N1 influenza hospitalizations in Maricopa County, Arizona, April 2009 to March 2010. *Comput Math Methods Med* 2012;2012.

**36.** Chowell G, Echevarria-Zuno S, Viboud C, et al. Epidemiological characteristics and underlying risk factors for mortality during the Autumn 2009 pandemic wave in Mexico. *PLoS ONE* 2012a;7 (7).

**37.** Choy J, Draper A, Ribbons E, et al. P245 Assessment of acute illness severity and radiological extent identify patients at heightened risk of developing major pneumonic progression in influenza A H1N1/2009 infection. *Thorax* 2010;65:A179-A180.

**38.** Chudasama RK, Patel UV, Verma PB. Hospitalizations associated with 2009 influenza A (H1N1) and seasonal influenza in Saurashtra region, India. *J Infect Dev Ctries* 2010;4:834-841.

**39.** Chudasama RK, Patel UV, Verma PB, et al. Clinico-epidemiological features of the hospitalized patients with 2009 pandemic influenza A (H1N1) virus infection in Saurashtra region, India (September, 2009 to February, 2010). *Lung India* 2011;28:11-16.

**40.** Chudasama RK, Patel UV, Verma PB, Patel RR, Patel PK. Clinical and epidemiological features among hospitalized patients of pandemic influenza A (H1N1) in Saurashtra region, India: a two wave comparison. *Int J Health Allied Sci* 2012;1:158-165.

**41.** Cocoros NM, Lash TL, Demaria A, Klompas M. Obesity as a risk factor for severe influenza-like illness. *Influenza Other Respir Viruses* 2014;8:25-32.

**42.** Cohen C, Moyes J, Tempia S, et al. Mortality amongst patients with influenza-associated severe acute respiratory illness, South Africa, 2009-2013. *PLoS ONE* 2015;10 (3).

**43.** Cortes Garcia M, Sierra Moros MJ, Santa-Olalla Peralta P, Hernandez-Barrera V, Jimenez-Garcia R, Pachon I. Clinical characteristics and outcomes of diabetic patients who were hospitalised with 2009 pandemic influenza A H1N1 infection. *Journal of Infection* 2012;64:218-224.

**44.** Creanga AA, Kamimoto L, Newsome K, et al. Seasonal and 2009 pandemic influenza A (H1N1) virus infection during pregnancy: A population-based study of hospitalized cases. *Am J Obstet Gynecol* 2011;204:S38-S45.

**45.** Crockett F, Mal H, Amazzough K, et al. H1N1 (2009) Influenza A infection in transplant recipient patients: A comparative study versus non-transplanted patients. *Revue de Pneumologie Clinique* 2011;67:199-208.

**46.** Cullen G, Martin J, O'Donnell J, et al. Surveillance of the first 205 confirmed hospitalised cases of pandemic H1N1 influenza in Ireland, 28 April - 3 October 2009. *Euro surveillance* 2009;14.

**47.** Cutter JL, Ang LW, Lai FYL, Subramony H, Ma S, James L. Outbreak of pandemic influenza A (H1N1-2009) in Singapore, May to September 2009. *Ann Acad Med Singapore* 2010;39:273-282.

**48.** D'Ortenzio E, Renault P, Jaffar-Bandjee MC, et al. A review of the dynamics and severity of the pandemic A(H1N1) influenza virus on Reunion island, 2009. *Clin Microbiol Infect* 2010;16:309-316.

**49.** Da Dalt L, Chillemi C, Cavicchiolo ME, et al. Pandemic influenza A (H1N1v) infection in pediatric population: a multicenter study in a North-East area of Italy. *Ital J Pediatr* 2011;37 (1).

**50.** Das RR, Sami A, Lodha R, et al. Clinical profile and outcome of swine flu in Indian children. *Indian Pediatrics* 2011;48:373-378.

**51.** de Muga MV, Monmany NT, Carretero SA, et al. Clinical features of influenza A H1N1 2009: a multicentre study. *Anales De Pediatria* 2011;75:6-12.

**52.** Dee S, Jayathissa S. Clinical and epidemiological characteristics of the hospitalised patients due to pandemic H1N1 2009 viral infection: Experience at Hutt Hospital, New Zealand. *New Zealand Medical Journal* 2010;123:45-53.

**53.** Del Rosal T, Baquero-Artigao F, Calvo C, et al. Pandemic H1N1 influenza-associated hospitalizations in children in Madrid, Spain. *Influenza Other Respir Viruses* 2011;5:e544-e551.

**54.** Delgado-Sanz C, Jiménez-Jorge S, López-Perea N, et al. Influenza surveillance in Spain. 2011-12 season (from week 40/2011 to week 20/2012). *Boletín Epidemiológico Semanal* 2012;20:153-167.

**55.** Delgado-Sanz C, Jiménez-Jorge S, Pozo F, et al. Influenza surveillance in Spain. Season 2012-2013 (from week 40/2012 to week 20/2013). *Boletín Epidemiológico Semanal* 2013;21:193-206.

**56.** Delgado-Sanz C, Jiménez-Jorge S, Pozo F, et al. Influenza surveillance in Spain, 2013-2014 season (from week 40 of 2013 until week 20 of 2014). *Boletín Epidemiológico Semanal* 2014;22:146-161.

**57.** Delgado-Sanz C, Jiménez-Jorge S, Pozo F, et al. Influenza surveillance in Spain. Report of the 2014-2015 influenza season, weeks 40/2014-08/2015 (September 29, 2014 to February 22, 2015). *Boletín Epidemiológico Semanal* 2015;23:1-11.

**58.** Desmoulins C, Michard-Lenoir AP, Naud J, Claudet I, Nouyrigat V, Cheron G. Clinical features and outcome of 2009 H1N1 influenza in the pediatric setting. Multicenter prospective study in the ED. *Archives de pediatrie* 2011;18:505-511.

**59.** Devaux I, Kreidl P, Penttinen P, et al. Initial surveillance of 2009 influenza A(H1N1) pandemic in the European Union and European economic area, April-September 2009. *Euro Surveillance* 2010;15:9.

**60.** Dolan GP, Myles PR, Brett SJ, et al. The comparative clinical course of pregnant and non-pregnant women hospitalised with influenza a(H1N1)pdm09 infection. *PLoS ONE* 2012;7 (8).

**61.** Echevarria-Zuno S, Mejia-Arangure JM, Mar-Obeso AJ, et al. Infection and death from influenza A H1N1 virus in Mexico: a retrospective analysis. *The Lancet* 2009;374:2072-2079.

**62.** Ergonul O, Alan S, Ak O, et al. Predictors of fatality in pandemic influenza A (H1N1) virus infection among adults. *BMC Infect Dis* 2014;14:317.

**63.** Fleege L, Hallberg E, Morin C, Danila R, Lynfield R. Novel H1N1 influenza hospitalizations: Minneapolis-St. Paul metropolitan area, 2008-2009. *Minnesota medicine* 2009;92:38-42.

**64.** Fuentes-Pacheco YC, Flores-Ruiz EM, Solorzano-Santos F, et al. Clinical characteristics of pediatric patients treated for influenza A (H1N1). The 2009 pandemic in Mexico. *Revista Medica del Instituto Mexicano del Seguro Social* 2014;52:S8-15.

**65.** Garcia MN, Philpott DC, Murray KO, et al. Clinical predictors of disease severity during the 2009-2010 A(HIN1) influenza virus pandemic in a paediatric population. *Epidemiol Infect* 2015;143:2939-2949.

**66.** Garnacho-Montero J, Gutierrez-Pizarraya A, Marquez JA, et al. Epidemiology, clinical features, and prognosis of elderly adults with severe forms of influenza A (H1N1). *Journal of the American Geriatrics Society* 2013;61:350-356.

**67.** Gasparini R, Amicizia D, Lai PL, Rossi S, Panatto D. Effectiveness of adjuvanted seasonal influenza vaccines (Inflexal V and Fluad) in preventing hospitalization for influenza and pneumonia in the elderly: a matched case-control study. *Human Vaccines Immunother* 2013;9:144-152.

**68.** Gauzere BA, Bussienne F, Bouchet B, et al. Severe cases of A(H1N1)v2009 infection in Reunion Island in 2009 and 2010. *Bulletin de la Societe de Pathologie Exotique* 2011;104:97-104.

**69.** Gentile A, Bakir J, Russ C, et al. Study of respiratory influenza A H1N1 Virus (pH1N1) in hospitalized children in the pandemic year. Experience in 34 centers in Argentina. *Archivos Argentinos de Pediatría* 2011;109:198-203.

**70.** Gilca R, de Serres G, Boulianne N, et al. Risk factors for hospitalization and severe outcomes of 2009 pandemic H1N1 influenza in Quebec, Canada. *Influenza Other Respir Viruses* 2011;5:247-255.

**71.** Godoy P, Rodes A, Alvarez J, et al. Characteristics of cases hospitalized for severe pandemic (H1N1) 2009 in Catalonia. *Revista Espanola de Salud Publica* 2011;85:81-87.

**72.** Goggin LS, Carcione D, Mak DB, et al. Chronic disease and hospitalisation for pandemic (H1N1) 2009 influenza in Indigenous and non-Indigenous Western Australians. *Commun Dis Intell* 2011;35:172-176.

**73.** Gonzalez-Candelas F, Astray J, Alonso J, et al. Sociodemographic factors and clinical conditions associated to hospitalization in influenza a (H1N1) 2009 virus infected patients in spain, 2009-2010. *PLoS ONE* 2012;7 (3).

**74.** Gonzalez R, Balasso V, Uriona S, et al. Patient characteristics and health care burden during the influenza A (H1N1) 2009 pandemic in the university Hospital Vall d'Hebron of Barcelona. *Enferm Infecc Microbiol Clin* 2011;29:593-600.

**75.** Goong HJ, Seo YR, Yoon LY, et al. Clinical and laboratory characteristics of pandemic influenza A/H1N1 2009 infection among patients with malignancy in Korea. *Infection and Chemotherapy* 2012;44:1-4.

**76.** Gordon CL, Johnson PDR, Permezel M, et al. Association between Severe Pandemic 2009 Influenza A (H1N1) Virus Infection and Immunoglobulin G(2) Subclass Deficiency. *Clin Infect Dis* 2010;50:672-678.

**77.** Grajeda Annca PF, Niño de Guzmán OF, Montoya Lizarraga M, Guevara Fernández Y. Risk factors associated to mortality by novel influenza A (H1N1) in Cusco-Peru. / Factores de riesgo asociados a mortalidad por nueva influenza A (H1N1) en la región Cusco-Perú. *Acta Medica Peruana* 2013;30:97-104.

**78.** Hanshaoworakul W, Simmerman JM, Narueponjirakul U, et al. Severe human influenza infections in Thailand: oseltamivir treatment and risk factors for fatal outcome. *PLoS ONE* 2009;4:e6051.

**79.** Hanslik T, Boelle PY, Flahault A. Preliminary estimation of risk factors for admission to intensive care units and for death in patients infected with A(H1N1)2009 influenza virus, France, 2009-2010. *PLoS Currents* 2010;2:RRN1150.

**80.** Harris PN, Dixit R, Francis F, et al. Pandemic influenza H1N1 2009 in north Queensland--risk factors for admission in a region with a large indigenous population. *Commun Dis Intell Q Rep* 2010;34:102-109.

**81.** Helferty M, Vachon J, Tarasuk J, Rodin R, Spika J, Pelletier L. Incidence of hospital admissions and severe outcomes during the first and second waves of pandemic (H1N1) 2009. *Canadian Medical Association Journal* 2010;182:1981-1987.

**82.** Hennessy TW, Bruden D, Castrodale L, et al. A case-control study of risk factors for death from 2009 pandemic influenza A(H1N1): Is American Indian racial status an independent risk factor? *Epidemiol Infect* 2016;144:315-324.

**83.** Hsann YM, Thang SP, Abdul Salam ZH, Yang Y, Sui-Leong Lim V, Yang KS. Clinical characteristics and outcomes of hospitalized patients with 2009 H1N1 influenza in a large acute care tertiary hospital, Singapore. *American Journal of Infection Control* 2011;39:e49-e51.

**84.** Husain EH, Alkhabaz A, Al-Qattan HY, Al-Shammari N, Owayed AF. Hospitalization patterns and outcomes of infants with influenza A(H1N1) in Kuwait. *J Infect Dev Ctries* 2012;6:632-636.

**85.** Ismail HIM, Tan KK, Lee YL, et al. Characteristics of children hospitalized for pandemic (H1N1) 2009, Malaysia. *Emerg Infect Dis* 2011;17:708-710.

**86.** Jain S, Kamimoto L, Bramley AM, et al. Hospitalized patients with 2009 H1N1 influenza in the United States, April-June 2009. *New England Journal of Medicine* 2009;361:1935-1944.

**87.** Javadi AA, Ataei B, Khorvash F, et al. Clinical features of novel 2009 influenza a (H1N1) infection in Isfahan, Iran. *Journal of Research in Medical Sciences* 2011;16:1550-1554.

**88.** Kark JD, Lebiush M, Rannon L. Cigarette smoking as a risk factor for epidemic A(H1N1) influenza in young men. *New England Journal of Medicine* 1982;307:1042-1046.

**89.** Kaya S, Yilmaz G, Arslan M, Oztuna F, Ozlu T, Koksal I. Predictive factors for fatality in pandemic influenza A (H1N1) virus infected patients. *Saudi Medical Journal* 2012;33:146-151.

**90.** Kelly PM, Kotsimbos T, Reynolds A, et al. FluCAN 2009: Initial results from sentinel surveillance for adult influenza and pneumonia in eight Australian hospitals. *Medical Journal of Australia* 2011;194:169-174.

**91.** Kobayashi M, Ohfuji S, Fukushima W, et al. Pediatric hospitalizations with influenza A infection during the 2009-2010 pandemic in five hospitals in Japan. *Pediatrics International* 2012;54:613-618.

**92.** Kusznierz G, Uboldi A, Sosa G, et al. Clinical features of the hospitalized patients with 2009 pandemic influenza A (H1N1) in Santa Fe, Argentina. *Influenza Other Respir Viruses* 2013;7:410-417.

**93.** Kwan-Gett TS, Baer A, Duchin JS. Spring 2009 H1N1 influenza outbreak in King County, Washington. *Disaster Medicine and Public Health Preparedness* 2009;3:S109-S116.

**94.** Labeaud AD, Wentworth B, Gildengorin G, Tam K, Guardia-Labar L, Petru A. Comparison of moderate and severe hospitalized pediatric 2009 H1N1 influenza cases. *Pediatr Infect Dis J* 2013;32:e90-e93.

**95.** Launes C, Garcia-Garcia JJ, Martinez-Planas A, et al. 2009 H1N1: Risk factors for hospitalization in a matched case-control study. *Eur J Pediatr* 2012;171:1127-1131.

**96.** Lee MC, Kim HY, Kong SG, et al. Clinical characteristics of pandemic influenza A (H1N1) 2009 pediatric infection in Busan and Gyeongsangnam-do: One institution. *Tuberc Respir Dis* 2012;72:493-500.

**97.** Lehners N, Geis S, Eisenbach C, Neben K, Schnitzler P. Changes in severity of influenza a(H1N1)pdm09 infection from pandemic to first postpandemic season, Germany. *Emerg Infect Dis* 2013;19:748-755.

**98.** Lenzi L, Silva LR, Mello AM, Grochocki MH, Pontarolo R. Factors related to death by the pandemic influenza A (H1N1) 2009 in patients treated with oseltamivir. *Revista brasileira de enfermagem* 2013;66:715-721.

**99.** Levy NS, Nguyen TQ, Westheimer E, Layton M. Disparities in the severity of influenza illness: a descriptive study of hospitalized and nonhospitalized novel H1N1 influenza-positive patients in New York City: 2009-2010 influenza season. *Journal of Public Health Management and Practice* 2013;19:16-24.

**100.** Li F, Chen G, Wang J, Liu H, Wu J. A case-control study on risk factors associated with death in pregnant women with severe pandemic H1N1 infection. *BMJ Open* 2012;2 (4).

**101.** Lim C, Ang LW, Tey SH, et al. Influenza A(H1N1)pdm09 infection in pregnant and non-pregnant women hospitalized in Singapore, May - December 2009. *Public Health* 2015;129:769-776.

**102.** Lopez-Medina E, Ardura MI, Siegel JD, Brock E, Sanchez PJ. 2009 influenza A in infants hospitalized at younger than 6 months. *Journal of Pediatrics* 2012;160:626-631.

**103.** Louie JK, Schechter R, Honarmand S, et al. Severe pediatric influenza in California, 2003-2005: Implications for immunization recommendations. *Pediatrics* 2006;117:e610-e618.

**104.** Louie JK, Acosta M, Winter K, et al. Factors associated with death or hospitalization due to pandemic 2009 influenza A(H1N1) infection in California. *JAMA* 2009;302:1896-1902.

**105.** Louie JK, Acosta M, Jamieson DJ, Honein MA. Severe 2009 H1N1 influenza in pregnant and postpartum women in California. *New England Journal of Medicine* 2010;362:27-35.

**106.** Louie JK, Jamieson DJ, Rasmussen SA. 2009 pandemic influenza A (H1N1) virus infection in postpartum women in California. *Am J Obstet Gynecol* 2011a;204:144.e141-144.e146.

**107.** Louriz M, Mahraoui C, Azzouzi A, et al. Clinical features of the initial cases of 2009 pandemic influenza A (H1N1) virus infection in an university hospital of Morocco. *International Archives of Medicine* 2010;3 (1).

**108.** Lowcock EC, Rosella LC, Foisy J, McGeer A, Crowcroft N. The social determinants of health and pandemic H1N1 2009 influenza severity. *American Journal of Public Health* 2012;102:e51-58.

**109.** Lynfield R, Davey R, Dwyer DE, et al. Outcomes of influenza A(H1N1)pdm09 virus infection: Results from two international cohort studies. *PLoS ONE* 2014;9 (7).

**110.** Ma H-Y, Wu JL, Lu CY, et al. Risk factors associated with severe influenza virus infections in hospitalized children during the 2013 to 2014 season. *J Microbiol Immunol Infect* 2016;49:387-393.

**111.** Martin ET, Archer C, McRoberts J, et al. Epidemiology of severe influenza outcomes among adult patients with obesity in Detroit, Michigan, 2011. *Influenza Other Respir Viruses* 2013;7:1004-1007.

**112.** Martinez E, Marcos M, Hoyo-Ulloa I, et al. Influenza A H1N1 in HIV-infected adults. *HIV Medicine* 2011;12:236-245.

**113.** Mata-Marín LA, Mata-Marín JA, Vásquez-Mota VC, et al. Risk factors associated with mortality in patients infected with influenza A/H1N1 in Mexico. *BMC Research Notes* 2015;8:432.

**114.** Mayoral Cortes JM, Fernandez JR, Diaz JP, et al. Infection by the pandemic virus (H1N1) 2009 in Andalusia. *Revista Espanola de Salud Publica* 2010;84:517-528.

**115.** Mayoral JM, Alonso J, Garin O, et al. Social factors related to the clinical severity of influenza cases in Spain during the A (H1N1) 2009 virus pandemic. *BMC Public Health* 2013;13:118.

**116.** McKenna JJ, Bramley AM, Skarbinski J, Fry AM, Finelli L, Jain S. Asthma in patients hospitalized with pandemic influenza A(H1N1)pdm09 virus infection-United States, 2009. *BMC Infect Dis* 2013;13 (1).

**117.** McMorrow ML, Wemakoy EO, Tshilobo JK, et al. Severe acute respiratory illness deaths in sub-Saharan Africa and the role of influenza: a case series from 8 countries. *J Infect Dis* 2015;212:853-860.

**118.** Meerhoff TJ, Simaku A, Ulqinaku D, et al. Surveillance for severe acute respiratory infections (SARI) in hospitals in the WHO European region - an exploratory analysis of risk factors for a severe outcome in influenza-positive SARI cases. *BMC Infect Dis* 2015;15:1.

**119.** Mehta AA, Anil Kumar V, Nair SG, Joseph FK, Kumar G, Singh SK. Clinical profile of patients admitted with swine-origin influenza a (H1N1) virus infection: An experience from a tertiary care hospital. *J Clin Diagn Res* 2013;7:2227-2230.

**120.** Mehta VK, Pooja S, Guleria RC, Ganju SA, Digvijay S, Anil K. Clinico-epidemiological profile, pandemic influenza A H1N1/2009 and seasonal influenza, August 2009-March 2013, Himachal Pradesh, India. *Indian Journal of Community Medicine* 2016;41:69-71.

**121.** Mickiene A, Daniuseviciute L, Vanagaite N, et al. Hospitalized adult patients with 2009 pandemic influenza a (H1N1) in Kaunas, Lithuania. *Medicina* 2011;47:11-18.

**122.** Miller AC, Subramanian RA, Safi F, Sinert R, Zehtabchi S, Elamin EM. Influenza A 2009 (H1N1) virus in admitted and critically ill patients. *Journal of Intensive Care Medicine* 2012;27:25-31.

**123.** Minnema BJ, Husain S, Mazzulli T, et al. Clinical characteristics and outcome associated with pandemic (2009) H1N1 influenza infection in patients with hematologic malignancies: A retrospective cohort study. *Leukemia and Lymphoma* 2013;54:1250-1255.

**124.** Miroballi Y, Baird JS, Zackai S, et al. Novel influenza A(H1N1) in a pediatric health care facility in New York City during the first wave of the 2009 pandemic. *Archives of Pediatrics and Adolescent Medicine* 2010;164:24-30.

**125.** Moghadami M, Kazeroni PA, Honarvar B, et al. Influenza A (H1N1) virus pandemic in Fars province: A report from southern Iran, July-December 2009. *Iranian Red Crescent Medical Journal* 2010;12:231-238.

**126.** Morgan OW, Bramley A, Fowlkes A, et al. Morbid obesity as a risk factor for hospitalization and death due to 2009 pandemic influenza A(H1N1) disease. *PLoS ONE* 2010;5:e9694.

**127.** Morris SK, Parkin P, Science M, et al. A retrospective cross-sectional study of risk factors and clinical spectrum of children admitted to hospital with pandemic H1N1 influenza as compared to influenza A. *BMJ Open* 2012;2 (2).

**128.** Mostaco-Guidolin LC, Towers SM, Buckeridge DL, Moghadas SM. Age distribution of infection and hospitalization among Canadian First Nations populations during the 2009 H1N1 pandemic. *American Journal of Public Health* 2013;103:e39-44.

**129.** Muller MP, McGeer AJ, Hassan K, Marshall J, Christian M, TIBDN. Evaluation of pneumonia severity and acute physiology scores to predict ICU admission and mortality in patients hospitalized for influenza. *PLoS ONE* 2010;5:e9563.

**130.** Myles PR, Semple MG, Lim WS, et al. Predictors of clinical outcome in a national hospitalised cohort across both waves of the influenza A/H1N1 pandemic 2009-2010 in the UK. *Thorax* 2012;67:709-717.

**131.** Nayman Alpat S, Usluer G, Ozgunes I, Doyuk Kartal E, Erben N. Clinical and epidemiologic characteristics of hospitalized patients with 2009 H1N1 influenza infection. *Influenza Research and Treatment* 2012;2012.

**132.** Nguyen-Van-Tam JS, Openshaw PJM, Hashim A, et al. Risk factors for hospitalisation and poor outcome with pandemic A/H1N1 influenza: United Kingdom first wave (May-September 2009). *Thorax* 2010;65:645-651.

**133.** Nickel KB, Marsden-Haug N, Lofy KH, et al. Age as an independent risk factor for intensive care unit admission or death due to 2009 pandemic influenza A (H1N1) virus infection. *Public Health Reports* 2011;126:349-353.

**134.** O'Riordan S, Barton M, Yau Y, Read SE, Allen U, Tran D. Risk factors and outcomes among children admitted to hospital with pandemic H1N1 influenza. *Canadian Medical Association Journal* 2010;182:39-44.

**135.** Oh WS, Lee SJ, Lee CS, et al. A prediction rule to identify severe cases among adult patients hospitalized with pandemic influenza A (H1N1) 2009. *Journal of Korean Medical Science* 2011;26:499-506.

**136.** Osoro EM, Munyua P, Muthoka P, et al. Hospitalized patients with pandemic (H1N1) 2009, Kenya. *Emerg Infect Dis* 2011;17:1744-1746.

**137.** Park YB, Kim C, Hwang YI, et al. Asthma and severity of the 2009 novel H1N1 influenza: A case-control study. *J Asthma* 2014;51:69-74.

**138.** Payet C, Lutringer-Magnin D, Cassier P, et al. Description of patients with confirmed influenza A(H1N1)pdm09 admitted to an intensive care unit and identification of severity risk factors. *Med Mal Infect* 2013;43:81-84.

**139.** Peralta PSO, Cortes-Garcia M, Vicente-Herrero M, et al. Risk factors for disease severity among hospitalised patients with 2009 pandemic influenza A (H1N1) in Spain, April - December 2009. *Euro Surveillance* 2010a;15:9-17.

**140.** Perez Navero JL, Rumbao Aguirre J, Correas Sanchez A, Saldana Garcia N, Munoz-Villanueva MC, Ibarra De La Rosa I. Clinical characteristics of patients with infection due to influenza A (H1N1) 2009 and critical pathology. *Anales de Pediatria* 2011;74:97-102.

**141.** Peters PJ, Skarbinski J, Louie JK, et al. HIV-infected hospitalized patients with 2009 pandemic influenza A (pH1N1)-United States, spring and summer 2009. *Clin Infect Dis* 2011;52:S183-S188.

**142.** Pinilla I, De Gracia MM, Quintana-Diaz M, Figueira JC. Radiological prognostic factors in patients with pandemic H1N1 (pH1N1) infection requiring hospital admission. *Emergency Radiology* 2011;18:313-319.

**143.** Placzek HE, Madoff LC. Association of age and comorbidity on 2009 influenza A pandemic H1N1-related intensive care unit stay in Massachusetts. *American Journal of Public Health* 2014;104:e118-e125.

**144.** Plessa E, Diakakis P, Gardelis J, Thirios A, Koletsi P, Falagas ME. Clinical features, risk factors, and complications among pediatric patients with pandemic influenza A (H1N1). *Clinical Pediatrics* 2010;49:777-781.

**145.** Poeppl W, Hell M, Herkner H, et al. Clinical aspects of 2009 pandemic influenza A (H1N1) virus infection in Austria. *Infection* 2011;39:341-352.

**146.** Poggensee G, Gilsdorf A, Buda S, et al. The first wave of pandemic influenza (H1N1) 2009 in Germany: From initiation to acceleration. *BMC Infect Dis* 2010;10 (no pagination).

**147.** Pramanick A, Rathore S, Peter JV, Moorthy M, Lionel J. Pandemic (H1N1) 2009 virus infection during pregnancy in south India. *International Journal of Gynecology and Obstetrics* 2011;113:32-35.

**148.** Puvanalingam A, Rajendiran C, Sivasubramanian K, Ragunanthanan S, Suresh S, Gopalakrishnan S. Case series study of the clinical profile of H1N1 swine flu influenza. *J Assoc Physicians India* 2011;59:14-16, 18.

**149.** Quach C, Piche-Walker L, Platt R, Moore D. Risk factors associated with severe influenza infections in childhood: implication for vaccine strategy. *Pediatrics* 2003;112:e197-201.

**150.** Quan-Tai X, Rui-Ping M, Feng Z, et al. Analysis on risk factors of influenza A (H1N1). *Infectious Diseases in Clinical Practice* 2011;19:187-191.

**151.** Ren YY, Yin YY, Li WQ, et al. Risk factors associated with severe manifestations of 2009 pandemic influenza A (H1N1) infection in China: a case-control study. *Virol J* 2013;10.

**152.** Rhim JW, Go EJ, Lee KY, et al. Pandemic 2009 H1N1 virus infection in children and adults: A cohort study at a single hospital throughout the epidemic. *International Archives of Medicine* 2012;5 (1).

**153.** Ribeiro AF, Pellini ACG, Kitagawa BY, et al. Risk factors for death from influenza A (H1N1)pdm09, State of Sao Paulo, Brazil, 2009. *PLoS ONE* 2015;10 (3).

**154.** Riera M, Payeras A, Marcos MA, et al. Clinical presentation and prognosis of the 2009 H1N1 influenza A infection in HIV-1-infected patients: A Spanish multicenter study. *AIDS* 2010;24:2461-2467.

**155.** Riquelme R, Riquelme M, Rioseco ML, et al. Characteristics of hospitalised patients with 2009 H1N1 influenza in Chile. *European Respiratory Journal* 2010;36:864-869.

**156.** Rodriguez-Rieiro C, Carrasco-Garrido P, Hernandez-Barrera V, et al. Pandemic influenza hospitalization in Spain (2009) Incidence, in-hospital mortality, comorbidities and costs. *Human Vaccines Immunother* 2012;8:443-447.

**157.** Rolland-Harris E, Vachon J, Kropp R, et al. Hospitalization of pregnant women with pandemic A(H1N1) 2009 influenza in Canada. *Epidemiol Infect* 2012;140:1316-1327.

**158.** Saleh P, Noshad H, Naghili B. Clinical manifestations of patients with novel H1N1 infection hospitalized in infectious disease ward, Sina Hospital, Tabriz, Iran. *Iranian Journal of Clinical Infectious Diseases* 2010;5:200-205.

**159.** Sansonetti P, Sali M, Fabbiani M, et al. Immune response to influenza A(H1N1)v in HIV-infected patients. *J Infect Dev Ctries* 2014;8:101-109.

**160.** Santillan Salas CF, Mehra S, Pardo Crespo MR, Juhn YJ. Asthma and severity of 2009 novel H1N1 influenza: A population-based case-control study. *J Asthma* 2013;50:1069-1076.

**161.** Satterwhite L, Mehta A, Martin GS. Novel findings from the second wave of adult pH1N1 in the United States. *Critical Care Medicine* 2010;38:2059-2061.

**162.** Schrag SJ, Shay DK, Gershman K, et al. Multistate surveillance for laboratory-confirmed, influenza-associated hospitalizations in children: 2003-2004. *Pediatr Infect Dis J* 2006;25:395-400.

**163.** Scriven J, McEwen R, Mistry S, et al. Swine flu: A Birmingham experience. *Clinical Medicine* 2009;9:534-538.

**164.** Serwint JR, Miller RM, Korsch BM. Influenza type A and B infections in hospitalized pediatric patients: Who should be immunized? *American Journal of Diseases of Children* 1991;145:623-626.

**165.** Sevencan F, Ertem MM, Ozcullu N, et al. Retrospective evaluation of laboratory-confirmed and recovered cases of influenza A(H1N1)v. *Turkish Journal of Medical Sciences* 2011;41:647-656.

**166.** Sharma CP, Sharma K, Sharma S, Kumar A, Gupta MK. Demographic correlates of swine flu cases attending a tertiary care hospital in Rajasthan. *Indian Journal of Preventive and Social Medicine* 2012;43:224-228.

**167.** Shimada T, Sunagawa T, Taniguchi K, et al. Description of hospitalized cases of influenza A(H1N1)pdm09 infection on the basis of the national hospitalized-case surveillance, 2009-2010, Japan. *Japanese Journal of Infectious Diseases* 2015;68:151-158.

**168.** Shlomai A, Nutman A, Kotlovsky T, Schechner V, Carmeli Y, Guzner-Gur H. Predictors of pandemic (H1N1) 2009 virus positivity and adverse outcomes among hospitalized patients with a compatible syndrome. *Israel Medical Association Journal* 2010;12:622-627.

**169.** Siau C, Tee A, Au V, et al. Influenza A H1N1 (2009): Clinical spectrum of disease among adult patients admitted to a regional hospital in Singapore. *Singapore Medical Journal* 2011;52:475-480.

**170.** Singh M, Sharma S. An epidemiological study of recent outbreak of influenza A H1N1 (swine flu) in western Rajasthan region of India. *Journal of Medical & Allied Sciences* 2013;3:48-52.

**171.** Singh M, Hakim A, Saini GL, Bhansali S. Epidemiology of pandemic influenza A (H1N1) 2009 in western Rajasthan, India: a retrospective study. *Scholars Journal of Applied Medical Sciences* 2014;2:142-146.

**172.** Singhal S, Sarda N, Arora R, Punia N, Jain A. Clinical profile & outcome of H1N1 infected pregnant women in a tertiary care teaching hospital of northern India. *The Indian Journal of Medical Research* 2014;139:454-458.

**173.** Skarbinski J, Jain S, Bramley A, et al. Hospitalized patients with 2009 pandemic influenza A (H1N1) virus infection in the United States--September-October 2009. *Clin Infect Dis* 2011;52:S50-S59.

**174.** Snacken R, Quinten C, Devaux I, et al. Surveillance of hospitalised severe cases of influenza A(H1N1)pdm09 and related fatalities in nine EU countries in 2010-2011. *Influenza Other Respir Viruses* 2012;6:e93-e96.

**175.** Socan M. Burden of hospitalizations for pandemic influenza in Slovenia. *Croatian Medical Journal* 2011;52:151-158.

**176.** Soub HA, Ibrahim AS, Maslamani MA, Al-Khal AL, Shaath S, Hamza NA. Epidemiology, risk factors, clinical features, and outcome of adult patients with severe pandemic A/H1N1/2009 influenza in Qatar: A retrospective study. *Infectious Diseases in Clinical Practice* 2014;22:339-343.

**177.** Staikowsky F, Vanhecke C, D'Andrea C, Souab A, Rakotoson R, Michault A. Outbreak of influenza pandemic virus A(H1N1) 2009 infections in the emergency department, Saint-Pierre, Reunion Island, July-September 2009. *Bulletin de la Societe de Pathologie Exotique* 2011;104:125-134.

**178.** Tabarsi P, Moradi A, Marjani M, et al. Factors associated with death or intensive care unit admission due to pandemic 2009 influenza A (H1N1) infection. *Annals of Thoracic Medicine* 2011;6:91-95.

**179.** Thompson DL, Jungk J, Hancock E, et al. Risk factors for 2009 pandemic influenza A (H1N1)-related hospitalization and death among racial/ethnic groups in New Mexico. *American Journal of Public Health* 2011;101:1776-1784.

**180.** Tutuncu EE, Ozturk B, Gurbuz Y, et al. Clinical characteristics of 74 pandemic H1N1 influenza patients from Turkey: Risk factors for fatality. *Saudi Medical Journal* 2010;31:993-998.

**181.** van't Klooster TM, Wielders CC, Donker T, et al. Surveillance of hospitalisations for 2009 pandemic influenza a(H1N1) in the Netherlands, 5 June - 31 December 2009. *Euro Surveillance* 2010;15:9-16.

**182.** Venkata C, Sampathkumar P, Afessa B. Hospitalized patients with 2009 H1N1 influenza infection: the Mayo Clinic experience. *Mayo Clin Proc* 2010;85:798-805.

**183.** Viasus D, Pano-Pardo JR, Pachon J, et al. Factors associated with severe disease in hospitalized adults with pandemic (H1N1) 2009 in Spain. *Clin Microbiol Infect* 2011;17:738-746.

**184.** Viasus D, Cordero E, Rodriguez-Bano J, et al. Changes in epidemiology, clinical features and severity of influenza A (H1N1) 2009 pneumonia in the first post-pandemic influenza season. *Clin Microbiol Infect* 2012;18:E55-E62.

**185.** Wada K, Nishiura H, Kawana A. An epidemiological analysis of severe cases of the influenza A (H1N1) 2009 virus infection in Japan. *Influenza Other Respir Viruses* 2010;4:179-186.

**186.** Walaza S, Tempia S, Dawood H, et al. Influenza virus infection is associated with increased risk of death amongst patients hospitalized with confirmed pulmonary tuberculosis in South Africa, 2010-2011. *BMC Infect Dis* 2015;15:1-16.

**187.** Weber-Carstens S, Goldmann A, Quintel M, et al. Extracorporeal lung support in H1N1 provoked acute respiratory failure: the experience of the German ARDS Network. *Deutsches Arzteblatt International* 2013;110:543-549.

**188.** Wie SH, So BH, Song JY, et al. A comparison of the clinical and epidemiological characteristics of adult patients with laboratory-confirmed influenza A or B during the 2011-2012 influenza season in Korea: a multi-center study. *PLoS ONE* 2013;8 (5).

**189.** Wu UI, Wang JT, Ho YC, Pan SC, Chen YC, Chang SC. Factors associated with development of complications among adults with influenza: A 3-year prospective analysis. *Journal of the Formosan Medical Association* 2012;111:364-369.

**190.** Xi XM, Xu YA, Jiang L, et al. Hospitalized adult patients with 2009 influenza A (H1N1) in Beijing, China: risk factors for hospital mortality. *BMC Infect Dis* 2010;10.

**191.** Xu C, Iuliano AD, Chen M, et al. Characteristics of hospitalized cases with influenza A (H1N1)pdm09 infection during first winter season of post-pandemic in China. *PLoS ONE* 2013;8 (2).

**192.** Yang L, Chan KP, Lee RSY, et al. Obesity and influenza associated mortality: Evidence from an elderly cohort in Hong Kong. *Preventive Medicine* 2013;56:118-123.

**193.** Yu H, Feng Z, Uyeki TM, et al. Risk factors for severe illness with 2009 pandemic influenza A (H1N1) virus infection in China. *Clin Infect Dis* 2011;52:457-465.

**194.** Zarychanski R, Stuart TL, Kumar A, et al. Correlates of severe disease in patients with 2009 pandemic influenza (H1N1) virus infection. *Canadian Medical Association Journal* 2010;182:257-264.

**195.** Zhang PJ, Cao B, Li XL, et al. Risk factors for adult death due to 2009 pandemic influenza A(H1N1) virus infection: a 2151 severe and critical cases analysis. *Chin Med J* 2013a;126:2222-2228.

**196.** Zhang Q, Ji W, Guo Z, Bai Z, MacDonald NE. Risk factors and outcomes for pandemic H1N1 influenza compared with seasonal influenza in hospitalized children in China. *Canadian Journal of Infectious Diseases and Medical Microbiology* 2012;23:199-203.

**197.** Zolotusca L, Jorgensen P, Popovici O, et al. Risk factors associated with fatal influenza, Romania, October 2009-May 2011. *Influenza Other Respir Viruses* 2014;8:8-12.
