# Supplementary material for: Risk factors for serious outcomes associated with influenza illness in high‐ versus low‐ and middle‐income countries: Systematic literature review and meta‐analysis
Source: Influenza Other Respir Viruses. 2017 Dec 2;12(1):22–9. doi: 10.1111/irv.12504 (PMC5818335; doi:10.1111/irv.12504)
Supplement: Supplementary file 10 [file IRV-12-22-s010.docx]

**Supplementary material: Search strategy for *risk factors for severe outcomes with influenza illness: systematic literature review and meta-analysis,* Coleman et al. 2017*.*Ovid MEDLINE(R) 1946 to March Week 2 2016**

| Influenza | influenza.mp. OR exp Influenza, Human/ OR exp Influenzavirus B/ OR influenzavirus.mp. OR exp Influenzavirus A/ OR (swine adj2 (flu or influenza)).mp. OR exp Orthomyxoviridae Infections/  OR severe acute respiratory infection*.mp OR SARI.mp or seasonal influenza.mp or pandemic influenza.mp. | 92,776 |
| --- | --- | --- |
| Outcomes | exp Mortality, Premature/ OR exp Fetal Mortality/ or exp Maternal Mortality/ or exp Child Mortality/ or exp Infant Mortality/ or exp Perinatal Mortality/ or mortalit*.mp. or exp Hospital Mortality/ or exp Mortality/ OR exp Fatal Outcome/ or fatal*.mp. or exp Abortion, Spontaneous/ or miscarriage*.mp OR exp Death/ or exp Sudden Infant Death/ or exp "Cause of Death"/ or exp Perinatal Death/ or death*.mp. or exp Maternal Death/ or exp Fetal Death/ or exp Infant Death/ OR casualty.mp  OR exp survival/ or disease progression/ or disease complication*.mp or exacerbate*.mp  OR hospitali*.mp OR exp Hospitalization/  OR exp Pneumonia/ or exp Pneumonia, Viral/ OR pneumonia.mp or acute respiratory distress.mp or exp Respiratory Distress Syndrome, Newborn/ or exp Respiratory Distress Syndrome, Adult/  OR morbid*.mp. or exp Morbidity/  OR shortness of breath.mp. OR hypoxia.mp. or exp Anoxia/ OR hypoxemia .mp or anoxia.mp or dyspnea.mp  OR intensive care*.mp. or exp Critical Care/ OR Intensive Care Units.mp OR ICU.mp  or ((intensive or critical) adj care*).mp. OR intensive care unit/ or ICU.mp OR critical illness/ or critical illness*.mp  or exp Ventilators, Mechanical/ or respiratory support*.mp OR ecmo.mp. or exp Extracorporeal Membrane Oxygenation/ or extracorporeal*.mp or ECLS.mp | 2,925,494 |
| Specific Disease/ Risk Groups | exp Risk Factors/ or risk*.mp. or exp Risk/ or risk factor*.mp  or determinant*.mp or predictor*.mp  OR exp epidemiology/ OR epidemiology.mp  Or (Increas* or high* adj2 probability or rate* or chance or likelihood).mp  Or (high* adj risk).mp or (at adj risk).mp | 13,167,779 |
| Poverty | Homelessness/ OR exp Poverty/ or Homeless Persons/ OR Vulnerable Populations/ OR disadvantage*.mp. OR dispossess*.mp. or destitute*.mp. or homeless*.mp. or impover*.mp. or (povert* or poor).mp. or (underprivileg* or under-privileg*).mp. OR (vulnerabl* adj (communit* or group* or individual* or famil* or population* or people* or person* or village*)).mp. OR (isolate* adj (communit* or group* or individual* or famil* or village* or population* or people* or person*)).mp.  OR exp Socioeconomic Factors/ OR exp Ethnic Groups/ OR Health status/ OR Minority Groups/ or Health Services, Indigenous/ or Medical Indigency/ or exp Social Welfare/ or exp Social Security/ or "Transients and Migrants" or aborig*.mp. or indigen*.mp. OR minorit*.mp  OR Crowding/ OR long-term care/ OR Institutionalization/  or developing countries/ or developing countr*.mp or (low adj income*).mp. OR ((low-income or low income) adj countr* or village* or population*).mp. OR ((middle-income or middle income) adj countr* or population* or village*).mp. OR (middle adj income*).mp. | 2,920,662 |
| Limits | [limit 1 and 2 and 3]  to humans | 8256 |
