# Supplementary material for: Risk factors for serious outcomes associated with influenza illness in high‐ versus low‐ and middle‐income countries: Systematic literature review and meta‐analysis
Source: Influenza Other Respir Viruses. 2017 Dec 2;12(1):22–9. doi: 10.1111/irv.12504 (PMC5818335; doi:10.1111/irv.12504)
Supplement: Supplementary file 11 [file IRV-12-22-s011.doc]

Coleman et al.

Supplementary material figure labels

Figure S1

Relative risk of severe outcome (intensive care and/or death) for patients hospitalized with laboratory confirmed influenza by chronic underlying disease status for studies conducted in low and middle income versus high income countries

Figure S1a

Funnel plot of pooled relative risk (pRR) by standard error of the logged pooled relative risk for severe outcome (intensive care and/or death) for patients hospitalized with laboratory confirmed influenza by chronic underlying disease status for studies conducted in low and middle income versus high income countries

NOTE: Two studies (Fleege et al. (2009) and Bassetti et al. (2010)) excluded from plot to allow for visualization of remaining studies. Both had high estimated RR and low weights. Both were from HIC.

Figure S2

Relative risk of severe outcome (intensive care and/or death) for patients hospitalized with laboratory confirmed influenza by chronic underlying disease status for studies of children compared with adults

NOTE: Studies combining results for children and adults were excluded

Figure S2a

Funnel plot of pooled relative risk (pRR) by standard error of the logged pooled relative risk for severe outcome (intensive care and/or death) for patients hospitalized with laboratory confirmed influenza by chronic underlying disease status for studies of children compared with adults

Figure S3

Relative risk of severe outcome (intensive care and/or death) for patients hospitalized with laboratory confirmed influenza by chronic underlying disease status for studies comparing pandemic and non-pandemic influenza seasons

NOTE: Studies combining results for pandemic and non-pandemic seasons were excluded

Figure S3a

Funnel plot of pooled relative risk (pRR) by standard error of the logged pooled relative risk for severe outcome (intensive care and/or death) for patients hospitalized with laboratory confirmed influenza by chronic underlying disease status for studies comparing pandemic and non-pandemic (or mixed pandemic and non-pandemic) influenza seasons

Two studies (Fleege et al. (2009) and Bassetti et al. (2010)) excluded from plot to allow for visualization of remaining studies. Both had high estimated RR and low weights. Both were from the 2009 pandemic.

Figure S4

Relative risk of severe outcome (intensive care and/or death) for patients hospitalized with laboratory confirmed influenza by presence of neurological and/or neuromuscular conditions comparing studies of children and adults

NOTE: Studies combining results for children and adults were excluded

Figure S4a

Funnel plot of pooled relative risk (pRR) by standard error of the logged pooled relative risk for severe outcome (intensive care and/or death) for patients hospitalized with laboratory confirmed influenza by presence of neurological and/or neuromuscular conditions comparing studies of children and adults
